# Sedentary behavior in mice induces metabolic inflexibility by suppressing skeletal muscle pyruvate metabolism

**DOI:** 10.1172/JCI167371

**Published:** 2024-04-23

**Authors:** Piyarat Siripoksup, Guoshen Cao, Ahmad A. Cluntun, J. Alan Maschek, Quentinn Pearce, Marisa J. Brothwell, Mi-Young Jeong, Hiroaki Eshima, Patrick J. Ferrara, Precious C. Opurum, Ziad S. Mahmassani, Alek D. Peterlin, Shinya Watanabe, Maureen A. Walsh, Eric B. Taylor, James E. Cox, Micah J. Drummond, Jared Rutter, Katsuhiko Funai

**Affiliations:** 1 Diabetes & Metabolism Research Center,; 2 Department of Physical Therapy and Athletic Training,; 3 Department of Biochemistry,; 4 Metabolomics Core Research Facility,; 5 Department of Nutrition & Integrative Physiology, and; 6 Molecular Medicine Program, University of Utah, Salt Lake City, Utah, USA.; 7 Fraternal Order of Eagles Diabetes Research Center, University of Iowa, Iowa City, Iowa, USA.; 8 Howard Hughes Medical Institute, University of Utah, Salt Lake City, Utah, USA.

**Keywords:** Metabolism, Mitochondria, Skeletal muscle

## Abstract

Carbohydrates and lipids provide the majority of substrates to fuel mitochondrial oxidative phosphorylation. Metabolic inflexibility, defined as an impaired ability to switch between these fuels, is implicated in a number of metabolic diseases. Here, we explore the mechanism by which physical inactivity promotes metabolic inflexibility in skeletal muscle. We developed a mouse model of sedentariness, small mouse cage (SMC), that, unlike other classic models of disuse in mice, faithfully recapitulated metabolic responses that occur in humans. Bioenergetic phenotyping of skeletal muscle mitochondria displayed metabolic inflexibility induced by physical inactivity, demonstrated by a reduction in pyruvate-stimulated respiration (*J*O_2_) in the absence of a change in palmitate-stimulated *J*O_2_. Pyruvate resistance in these mitochondria was likely driven by a decrease in phosphatidylethanolamine (PE) abundance in the mitochondrial membrane. Reduction in mitochondrial PE by heterozygous deletion of phosphatidylserine decarboxylase (PSD) was sufficient to induce metabolic inflexibility measured at the whole-body level, as well as at the level of skeletal muscle mitochondria. Low mitochondrial PE in C2C12 myotubes was sufficient to increase glucose flux toward lactate. We further implicate that resistance to pyruvate metabolism is due to attenuated mitochondrial entry via mitochondrial pyruvate carrier (MPC). These findings suggest a mechanism by which mitochondrial PE directly regulates MPC activity to modulate metabolic flexibility in mice.

## Abstract 2

Physical inactivity reduces mitochondrial phosphatidylethanolamine to suppress skeletal muscle pyruvate metabolism and induce metabolic inflexibility in mice.

## Introduction

Chronic physical inactivity increases all-cause mortality by 30%, accounting for one death every 44 seconds (1–4). Sedentary behavior exacerbates the risk for many chronic diseases, such as type 2 diabetes and cardiovascular diseases (5–7). Systemic metabolic disturbances induced by inactivity are likely largely responsible for the pathogenesis of these conditions (7, 8). Described often as “metabolic inflexibility,” long-term sedentariness impairs the ability to switch between glucose and fatty acids to fuel ATP synthesis (9, 10). Metabolic inflexibility that occurs with physical inactivity is primarily driven by the suppression of glucose metabolism in skeletal muscle. Disuse likely directly drives the metabolic reprogramming to attenuate glycolytic flux to mitochondria in the absence of elevated energy demand. The mechanism by which skeletal muscle mitochondrial metabolism adapts to chronic disuse is not well understood.

Our understanding of the underlying molecular processes that drive inactivity-induced metabolic inflexibility has been limited partly by the lack of appropriate preclinical models of human sedentary behavior (11). Traditional murine models of muscle disuse or physical inactivity, such as hind-limb unloading, cast immobilization, and denervation models, are well suited to study muscle atrophy, but they do not phenocopy the systemic and skeletal muscle metabolic adaptations observed in humans (11, 12). To address this important methodological gap, we adapted a novel mouse model of inactivity, small mouse cage (SMC) (13, 14), that more reliably induces metabolic perturbations in skeletal muscle observed with sedentary behavior. This model has now enabled us to more rigorously investigate the interplay between mitochondrial energetics and metabolic inflexibility in the context of physical inactivity.

Previously, we identified mitochondrial phosphatidylethanolamine (PE) to be an important regulator of mitochondrial oxidative phosphorylation (OXPHOS) in skeletal muscle that is induced by exercise training and suppressed with hind-limb unloading (15). PE is highly concentrated in the inner mitochondrial membrane (IMM) and is autonomously synthesized by phosphatidylserine decarboxylase (PSD) (16, 17). In mammalian systems, nearly all PE is synthesized in the IMM by PSD and exported to other regions of the cell, while the PE generated by the CDP-ethanolamine pathway in the endoplasmic reticulum (ER) does not translocate to mitochondria (18, 19). Human mutation in the *PISD* gene, which encodes for the PISD enzyme, causes mitochondrial disease (20–22). We have previously shown that skeletal muscle–specific deletion of PSD (homozygous knockout) in mice is lethal owing to robust atrophy and weakness of the diaphragm muscle (15). The consequence of a more modest reduction of mitochondrial PE, such as that occurring with sedentariness, is unknown. Importantly, muscle phospholipid composition, particularly low PE, has been linked to metabolic inflexibility in humans (23–26).

In this study, we implicate reduced muscle mitochondrial PE as the driving force behind inactivity-induced metabolic inflexibility. SMC intervention modestly lowered mitochondrial PE, concomitantly with reduced glucose metabolism. We then recapitulated moderate reductions in mitochondrial PE using a skeletal muscle–specific heterozygous knockout of PSD (PSD-Mhet). Unlike their homozygous counterparts, mice with heterozygous deletion of PSD demonstrated modest systemic and skeletal muscle phenotypes that resembled many metabolic shifts found with the SMC intervention.

## Results

### SMC housing induces metabolic inflexibility in male but not female mice.

**Figure 1:**
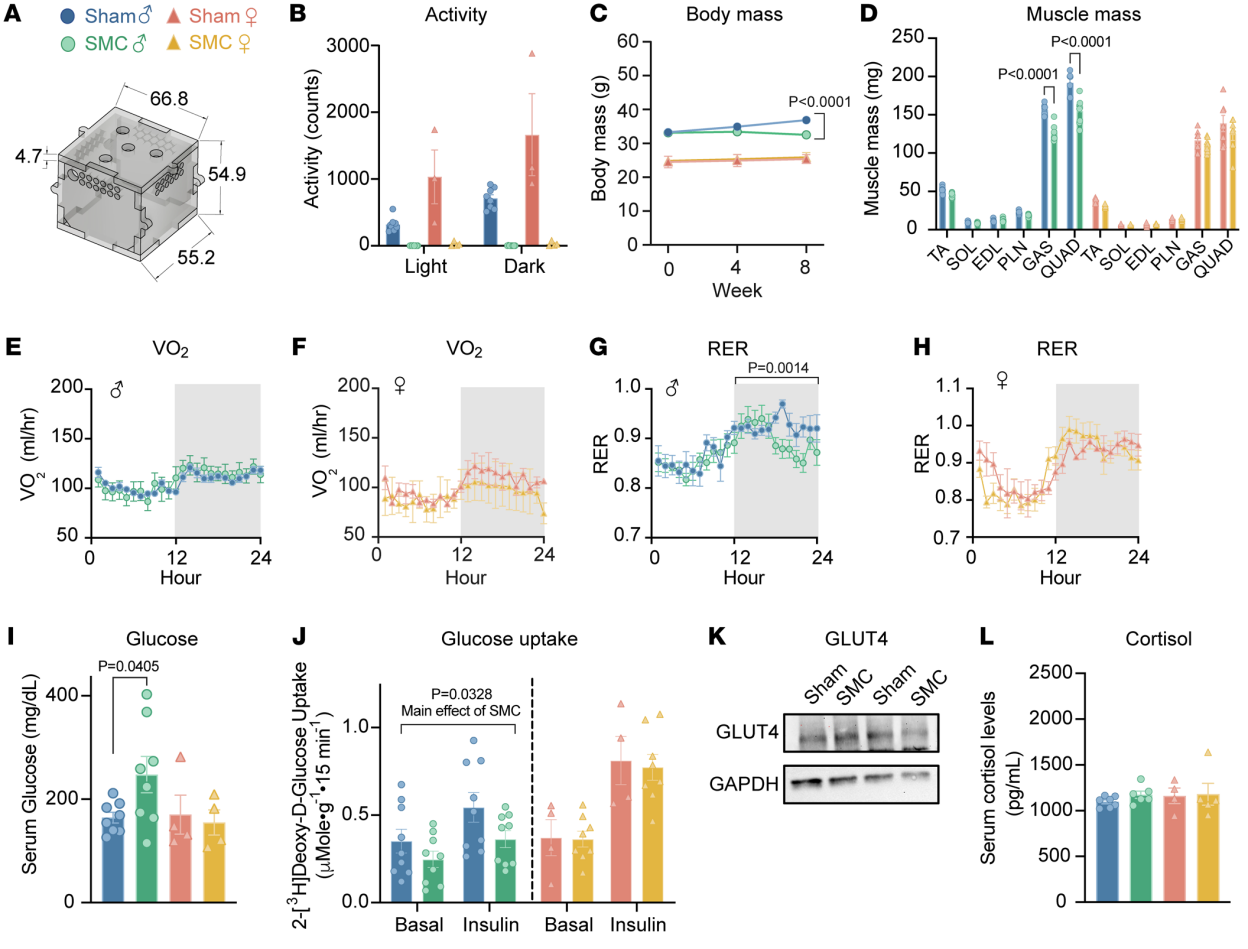
SMC housing induces metabolic inflexibility in male but not female mice. (**A**) Small mouse cage (SMC) schematic. (**B**) Activity counts of sham and SMC mice via indirect calorimetry (*n* = 8 per group). (**C**) Body mass time course (*n* = 6–14 per group). (**D**) Skeletal muscle tissue mass (*n* = 7–8 per group). (**E**) Absolute VO_2_ of male sham and SMC mice via indirect calorimetry (*n* = 8–9 per group). (**F**) Absolute VO_2_ of female sham and SMC mice via indirect calorimetry (*n* = 3–4 per group). (**G**) Respiratory exchange ratio (RER) of male sham and SMC mice (*n* = 8–9 per group). (**H**) RER of female sham and SMC mice (*n* = 3–4 per group). (**I**) Fasting serum glucose levels of sham and SMC mice (*n* = 4–8 per group). (**J**) [^3^H]2-Deoxyglucose glucose uptake in soleus muscles of male and female sham and SMC mice with or without 200 μU/mL of insulin (*n* = 4–9 per group). (**K**) GLUT4 and GAPDH protein abundance in gastrocnemius muscles. (**L**) Circulating cortisol levels from male and female sham and SMC mice (*n* = 4–7 per group). TA, tibialis anterior; SOL, soleus; EDL, extensor digitorum longus; PLN, plantaris; GAS, gastrocnemius; QUAD, quadriceps. Data represent mean ± SEM. *P* values generated by 2-tailed, equal-variance, Student’s *t* test (**D**) or by 2-way ANOVA with Tukey’s post hoc test (**B**, **C**, **E**–**H**, and **I**).

Sedentary behavior promotes systemic and skeletal muscle metabolic inflexibility in humans (7, 27). In contrast, commonly utilized models of disuse in mice such as hind-limb unloading increase skeletal muscle glucose uptake (Supplemental Figure 1A; supplemental material available online with this article; https://doi.org/10.1172/JCI167371DS1). To better model the metabolic disturbances observed in human inactivity, we developed a mouse model of physical inactivity using SMC (Figure 1A). Male and female wild-type C57BL/6J mice were ambulatory or were subjected to 8 weeks of SMC housing that substantially restricted gross spontaneous movement (Figure 1B). Body mass, lean mass, and individual muscle masses were significantly reduced in male mice and not in female mice (Figure 1, C and D, and Supplemental Figure 1B). In contrast, SMC intervention did not alter adiposity in either sex, although there was a trend for greater adipose tissue masses only in female mice (Supplemental Figure 1, C and D). To evaluate the effects of reduced activity on metabolic flexibility, mice underwent indirect calorimetry for measurements of whole-body O_2_ consumption (VO_2_) and respiratory exchange ratio (RER). VO_2_ was not influenced with SMC in both sexes (Figure 1, E and F), consistent with findings that changes in physical activity do not drive changes in total daily energy expenditure (28). RER is an indicator of systemic substrate preference, where a value of 1.0 signifies a 100% reliance on carbohydrates, whereas a value of 0.7 indicates a 100% reliance on lipids. Mice rely more on lipids during the light cycle when they are asleep and shift to carbohydrate utilization during the dark cycle when they are active or eating. Notably, while SMC induced metabolic inflexibility in male mice, female mice demonstrated normal metabolic flexibility (Figure 1, G and H, and Supplemental Figure 1, E and F). Specifically, SMC reduced the ability of male mice to shift to carbohydrate usage during the dark cycle. Further, consistent with attenuated systemic glucose metabolism, SMC intervention elevated fasting serum glucose in male mice (Figure 1I) without alterations in serum insulin levels (Supplemental Figure 1G).

To examine glucose metabolism in skeletal muscle, we excised soleus muscles from male and female sham or SMC mice for the measurement of ex vivo 2-deoxyglucose uptake. Congruent with systemic metabolic inflexibility, SMC intervention reduced glucose uptake in both basal and insulin-stimulated conditions in males, but not in females (Figure 1J). These changes in muscle glucose uptake occurred in the absence of changes in total GLUT4 content (Figure 1K). These findings are consistent with the hypothesis that reduced skeletal muscle glucose metabolism drives systemic metabolic inflexibility induced by SMC. It is noteworthy that male mice became metabolically inflexible despite no increases in adiposity (Supplemental Figure 1, C and D). Metabolic inflexibility also occurred independently of increases in food intake or serum cortisol levels (Figure 1L and Supplemental Figure 1H). Glucose tolerance was not different between sham and SMC groups in male or female mice (Supplemental Figure 1, I–L). These results are consistent with findings from human bed rest studies (29), where no differences in systemic glucose tolerance were found with bed rest in lean healthy young males. We interpret these findings to mean that reduced skeletal muscle glucose uptake precedes robust changes in systemic glucose metabolism, which can be detected with RER but not with glucose tolerance test at this particular time point.

**Figure 2:**
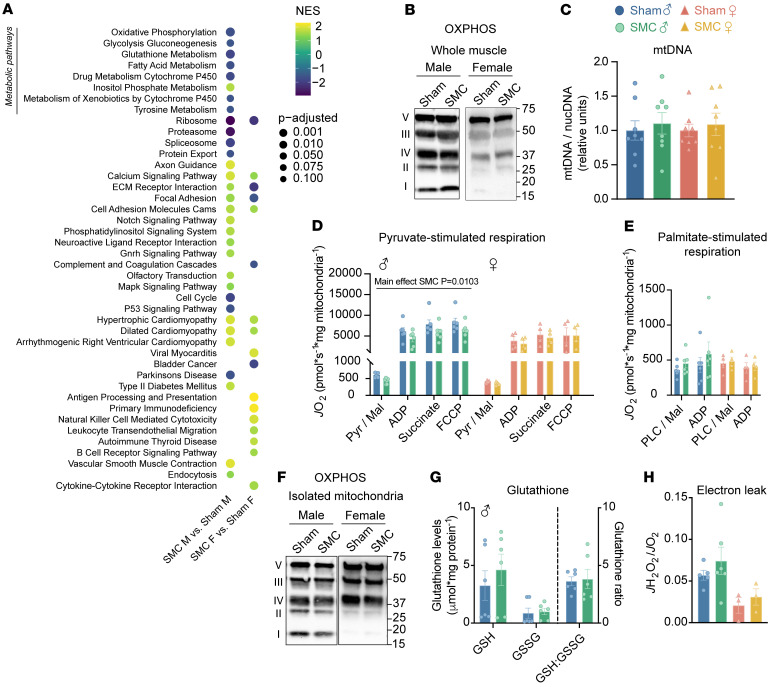
SMC housing reduces pyruvate-dependent respiration without altering palmitate-stimulated respiration. (**A**) Dot plot representing gene set enrichment analysis (GSEA) pathway analysis (Kyoto Encyclopedia of Genes and Genomes) of differentially expressed genes (FDR < 0.05) in skeletal muscle of sham and SMC mice. Normalized enrichment scores are represented by a darker color (negatively enriched) and lighter color (positively enriched), while a larger dot diameter indicates a smaller adjusted *P* value. Dot plot was generated with R Studio. (**B**) Representative Western blot of respiratory complexes (I–V) of whole muscle tissue of sham and SMC mice (*n* = 3–4 per group). (**C**) Ratio of nuclear to mitochondrial DNA in gastrocnemius muscle (*n* = 8 per group). (**D**) O_2_ utilization in isolated mitochondria from gastrocnemius muscle measured in the presence of 2 mM ADP, 0.5 mM malate (Mal), 5 mM pyruvate (Pyr), 10 mM succinate, 1 μM carbonyl cyanide-*p*-trifluoromethoxyphenylhydrazone (FCCP) of sham and SMC mice (*n* = 4–6 per group). (**E**) O_2_ utilization in isolated mitochondria measured in the presence of 2 mM ADP (adenosine diphosphate), 0.5 mM malate, 0.02 mM palmitoyl-l-carnitine (PLC) (*n* = 4–6 per group). (**F**) Representative Western blot of respiratory complex proteins in isolated muscle mitochondria of sham and SMC mice (*n* = 5–6 per group). (**G**) Reduced (GSH) and oxidized (GSSG) glutathione levels in plantaris muscle of sham and SMC mice (*n* = 6 per group). (**H**) Electron leak (*J*H_2_O_2_/O_2_) with succinate in isolated muscle mitochondria from gastrocnemius muscle of sham and SMC mice (*n* = 3–6 per group). Data represent mean ± SEM. *P* values generated by 2-way ANOVA with Tukey’s post hoc test (**C**–**E**, **G**, and **H**).

We sought to capitalize on the sexually dimorphic response to explore the mechanism by which SMC induces skeletal muscle metabolic inflexibility only in male mice. RNA sequencing of gastrocnemius muscles followed by Kyoto Encyclopedia of Genes and Genomes pathway analysis revealed similarities and differences in gene set enrichment in a number of pathways between males and females (Figure 2A). The ribosomal pathway was among the most negatively enriched categories with both sexes, consistent with the notion that inactivity decreases muscle protein synthesis (30). Notably, metabolic pathways were reduced in males but not in females, suggesting that metabolic reprogramming induced by SMC may be unique to males. Given the central role of mitochondria in these pathways, we further examined the effects of SMC on skeletal muscle mitochondria.

### SMC housing reduces pyruvate-dependent respiration without altering palmitate-stimulated respiration.

Previous reports suggest that reduced muscle mitochondrial content can potentially drive metabolic inflexibility induced by inactivity (7, 29). However, our SMC intervention did not alter mitochondrial density in skeletal muscle regardless of sex (Figure 2, B and C, and Supplemental Figure 2A), indicating that lower mitochondrial content is not necessary for inactivity-induced suppression of skeletal muscle glucose metabolism (31). Combined with data from RNA sequencing showing that expression of genes in mitochondrial pathways is reduced with SMC, the findings suggests that the influence of physical inactivity on mitochondria can be more nuanced. To this end, we further examined respiratory function per unit of mitochondria isolated from gastrocnemius muscle, which represents a muscle with mixed fiber-type composition. High-resolution respirometry experiments showed that SMC diminished respiration (*J*O_2_) driven by pyruvate in male but not in female mice (Figure 2D), consistent with the notion that metabolic inflexibility is driven by mitochondria’s ability to accept glycolytic substrates. Strikingly, there was no difference in *J*O_2_ fueled by palmitate (Figure 2E), indicating that the reduced ability of mitochondria to accept substrates is limited to glycolytic substrates. Moreover, these changes occurred independently of changes in OXPHOS protein abundances per unit of mitochondria (Figure 2F and Supplemental Figure 2B).

Some studies indicate that mitochondrial electron leak can promote oxidative stress to suppress glucose metabolism (32). Multiple laboratories, including our group’s, have reported that traditional models of disuse promote oxidative stress in skeletal muscle (33, 34). However, our SMC intervention did not alter the ratio of reduced to oxidized glutathione (GSH/GSSG) (Figure 2G) nor reactive lipid aldehydes such as 4-hydroxynonenal (Supplemental Figure 2, C and D), demonstrating that physical inactivity induced by SMC does not promote oxidative stress. Using high-resolution fluorometry, we further confirmed mitochondrial electron leak (*J*H_2_O_2_/*J*O_2_) to be unaltered with the SMC intervention (Figure 2H). These findings are consistent with results from human bed rest studies (35, 36), ruling out oxidative stress as a mechanism by which SMC intervention suppresses skeletal muscle glucose metabolism.

**Figure 3:**
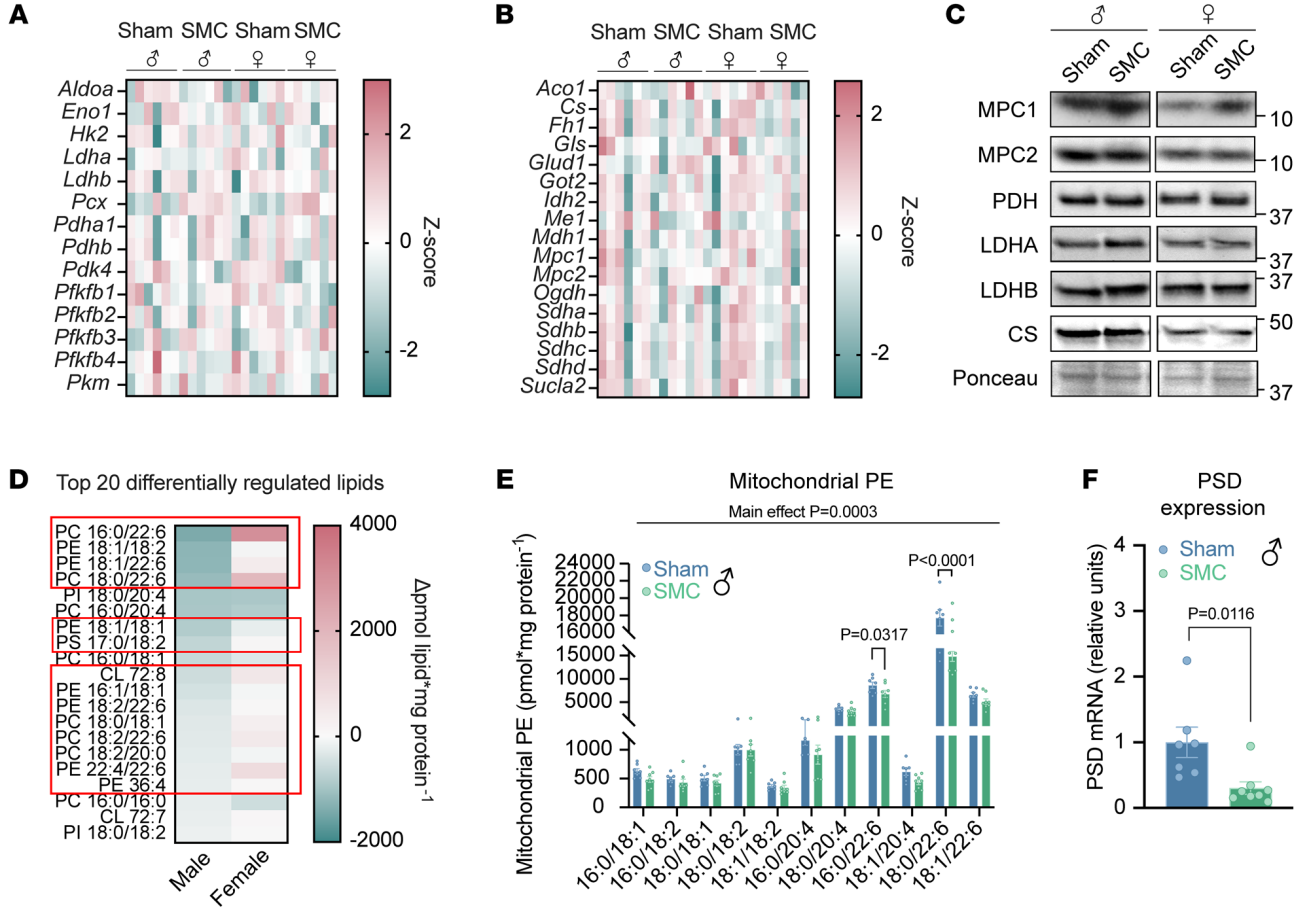
Physical inactivity by SMC housing alters skeletal muscle membrane lipid composition. (**A**) Heatmap of glycolytic genes in sham and SMC mice (*n* = 6 per group). (**B**) Heatmap of tricarboxylic acid (TCA) cycle genes in sham and SMC mice (*n* = 6 per group). (**C**) Representative Western blots of glycolytic/TCA genes in sham and SMC mice (*n* = 5–7 per group). (**D**) Top 20 differentially regulated skeletal muscle (gastrocnemius) mitochondrial lipids between SMC and sham mice (*n* = 7–8 per group). The red boxes highlight the lipids whose change in abundance is unique to male mice. (**E**) Skeletal muscle mitochondrial PE species (gastrocnemius) of sham and SMC mice (*n* = 8 per group). (**F**) Skeletal muscle phosphatidylserine decarboxylase (PSD) mRNA levels of sham and SMC mice (*n* = 7–8 per group). MPC1, mitochondrial pyruvate carrier complex 1; MPC2, mitochondrial pyruvate carrier complex 2; PDH, pyruvate dehydrogenase; LDHA, lactate dehydrogenase isoform A; LDHB, lactate dehydrogenase isoform B; CS, citrate synthetase. Data represent mean ± SEM. *P* values generated by 2-tailed, equal-variance, Student’s *t* test (**F**) or by 2-way ANOVA with Tukey’s post hoc test (**A**, **B**, **D**, and **E**).

What is the mechanism by which physical inactivity selectively suppresses mitochondrial pyruvate metabolism in skeletal muscle? SMC intervention had no effect on mRNA levels of pyruvate/glucose metabolism and TCA cycle, nor on protein levels of enzymes of pyruvate metabolism (Figure 3, A–C, and Supplemental Figure 2E), indicating that reductions in pyruvate oxidation cannot be attributed to changes in these enzymes. SMC also did not decrease enzyme activities of skeletal muscle pyruvate dehydrogenase, phosphofructokinase, or citrate synthase in male mice (Supplemental Figure 2, F–H). Mitochondrial membrane lipids are known to alter the activity of mitochondrial enzymes in multiple tissues, including skeletal muscle (15, 36). Particularly, disuse induced by hind-limb unloading reduces mitochondrial PE in skeletal muscle (15). Thus, we examined the effect of SMC housing on the skeletal muscle mitochondrial lipidome. Using liquid chromatography–tandem mass spectrometry, we quantified a total of 243 lipids from isolated mitochondria of gastrocnemius muscles of sham and SMC mice. Analyses of these lipids revealed a trend for an overall reduction (*P =* 0.118) in total phospholipid abundance with SMC in males but not in females (*P =* 0.789) (Supplemental Figure 3A). Seventy-three of the 243 lipids were significantly downregulated with SMC in male mice (zero upregulated lipids) (Supplemental Figure 3B), while only 2 reached statistical significance in female mice (Supplemental Figure 3C). Among these lipids, mitochondrial PE was most robustly disproportionately downregulated in male SMC mice (Figure 3, D and E, and Supplemental Figure 3D), consistent with our previous findings regarding PE with hind-limb unloading (15). Reduced PE with SMC was specific to mitochondria and was not reflected in total cellular PE content (Supplemental Figure 3, E and F). The observation that SMC reduced mitochondrial PE in only male mice is likely due to males’ tending to have greater mitochondrial PE to begin with (Supplemental Figure 3G). In turn, this may contribute to the lack of an SMC-induced phenotype in female mice. Mitochondrial PE is almost exclusively generated by the enzyme PSD from phosphatidylserine (PS). SMC did not influence the abundance of mitochondrial PS (nor an alternate PE precursor, lyso-PE) (Supplemental Figure 3, H–K). Nevertheless, SMC substantially reduced the abundance of PSD mRNA in skeletal muscle (Figure 3F). Thus, we proceeded to investigate the role that mitochondrial PE may play in metabolic inflexibility induced by physical inactivity.

### Muscle PSD haploinsufficiency makes mice more susceptible to inactivity-induced metabolic inflexibility.

**Figure 4:**
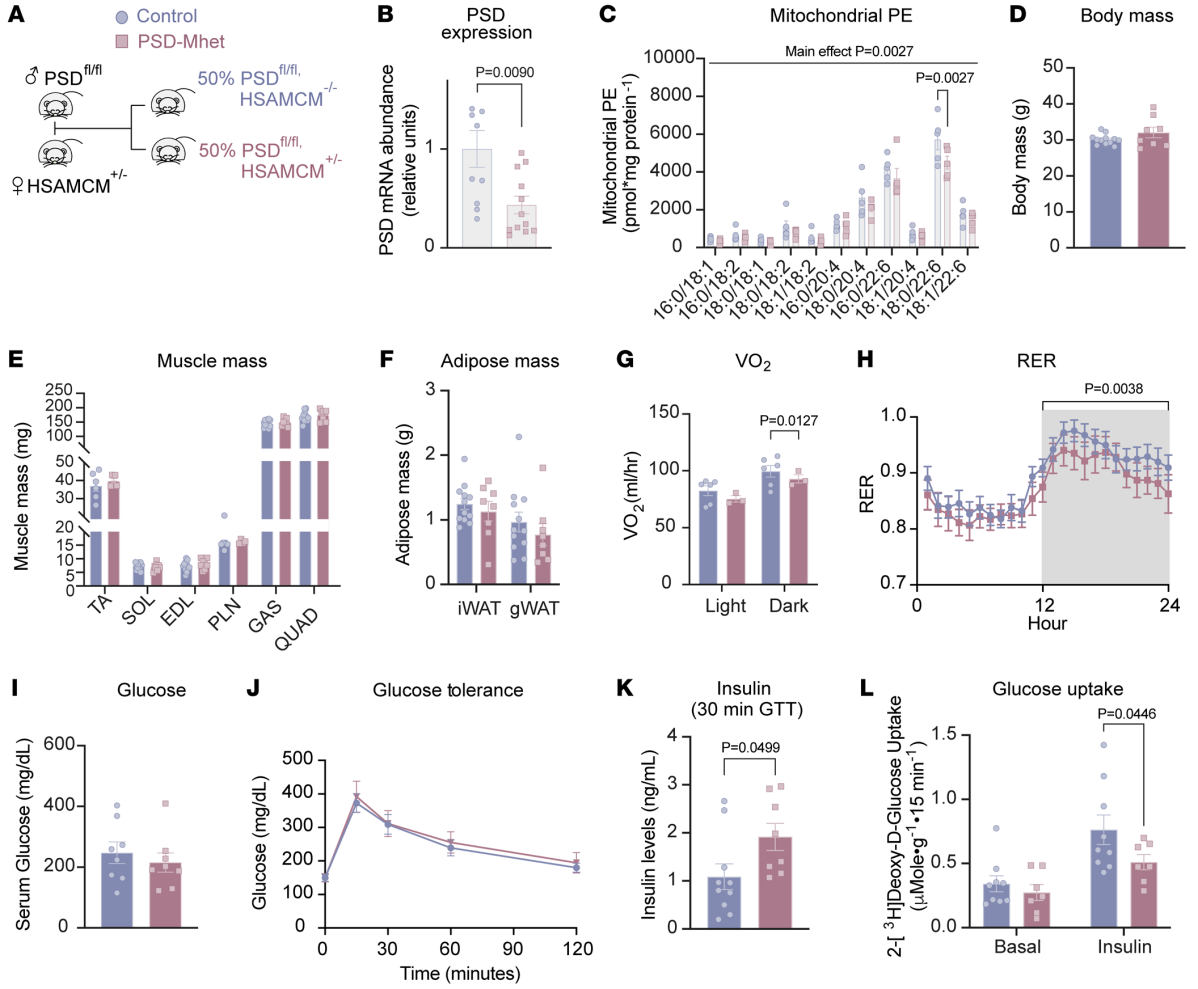
Muscle PSD haploinsufficiency increases susceptibility of mice to inactivity-induced metabolic inflexibility. (**A**) Mouse breeding schematic. (**B**) PSD mRNA levels of sham Cre control and PSD-Mhet (PSD muscle-specific heterozygous knockout) mice (*n* = 11–12 per group). (**C**) Muscle mitochondrial PE levels (from gastrocnemius muscles) of sham control and PSD-Mhet mice (*n* = 5 per group). (**D**) Body mass of SMC control and SMC PSD-Mhet mice after 8 weeks of reduced activity (*n* = 8–12 per group). (**E** and **F**) Skeletal muscle (**E**) and adipose masses (**F**) after SMC intervention (*n* = 8–12 per group). (**G**) Absolute VO_2_ via indirect calorimetry (*n* = 3–6 per group). (**H**) RER (*n* = 8–11 per group). (**I**) Serum glucose levels (*n* = 8 per group). (**J**) Glucose tolerance test (GTT) performed around week 7 of SMC intervention (*n* = 8–13 per group). (**K**) Serum insulin levels taken at the 30-minute time point during the GTT (*n* = 8–10 per group). (**L**) [^3^H]2-Deoxyglucose uptake in soleus muscles after 8 weeks of SMC (*n* = 7–9 per group). TA, tibialis anterior; SOL, soleus; EDL, extensor digitorum longus; PLN, plantaris; GAS, gastrocnemius; QUAD, quadriceps; iWAT, inguinal white adipose tissue; gWAT, gonadal white adipose tissue. All data are from male control and PSD-Mhet mice. Data represent mean ± SEM. *P* values generated by 2-tailed, equal-variance, Student’s *t* test (**B**, **D**, **I**, and **K**) or by 2-way ANOVA with Tukey’s post hoc test (**C**, **E**–**H**, **J**, and **L**).

Previously, we demonstrated that homozygous deletion of muscle PSD causes lethality due to metabolic and contractile failure in the diaphragm muscle (15). Homozygous deletion promotes a reduction in mitochondrial PE that is far more robust in magnitude compared with changes in mitochondrial PE observed with SMC. To model a more modest reduction in skeletal muscle mitochondrial PE, we studied mice with tamoxifen-inducible muscle-specific PSD heterozygous deletion (PSD-Mhet; PSD^fl/fl^ and HSA-MerCreMer^+/–^) (Figure 4A). As designed, skeletal muscle from PSD-Mhet mice had reduced PSD mRNA abundance compared with controls (PSD-Mhet; PSD^fl/fl^ and HSA-MerCreMer^–/–^) (Figure 4B), as well as modest depletions in some species of mitochondrial PE (Figure 4C and Supplemental Figure 4A) in male mice. Heterozygous deletion of PSD was not sufficient to significantly reduce mitochondrial PE in female mice. Unlike the PSD-homozygous-knockout mice, PSD-Mhet appeared normal and healthy under unstressed conditions (Supplemental Figure 4, B–I). Under sham conditions, PSD haploinsufficiency was found to impair mitochondrial energetics under pyruvate-stimulated conditions (Supplemental Figure 4J), but not under palmitate-induced conditions (Supplemental Figure 4K). However, PE deficiency was not adequate to attenuate skeletal muscle 2-deoxyglucose uptake in soleus muscles.(Supplemental Figure 4L). Taken together, these results suggest sufficiency for PE to alter maximal skeletal muscle respiration, but insufficiency to cause metabolic inflexibility under sham conditions.

We placed control and PSD-Mhet male mice on 8 weeks of SMC to study their systemic and skeletal muscle metabolism. Muscle PSD haploinsufficiency did not influence body mass, body composition, food intake, serum cortisol, or masses of skeletal muscle and adipose tissues (Figure 4, D–F, and Supplemental Figure 4, M–O). Indirect calorimetry of these mice showed a slight reduction in whole-body VO_2_ in PSD-Mhet compared with controls (Figure 4G), which was not explained by changes in physical activity (both virtually undetectable with SMC). Consistent with our hypothesis that low mitochondrial PE may drive metabolic inflexibility, RER data revealed suppression of glucose metabolism during dark cycle in PSD-Mhet mice compared with control mice (Figure 4H and Supplemental Figure 4P). Neither fasting glucose nor glucose tolerance was different between the groups (Figure 4, I and J). However, circulating insulin levels at the 30-minute time point of the glucose tolerance test was higher in PSD-Mhet compared with controls (Figure 4K), suggesting that PSD haploinsufficiency may require greater circulating insulin to stimulate muscle glucose metabolism. Indeed, skeletal muscle glucose uptake was attenuated in PSD-Mhet mice compared with control mice (Figure 4L). Collectively, these results suggest that muscle PE deficiency may impair skeletal muscle glucose metabolism to promote metabolic inflexibility.

**Figure 5:**
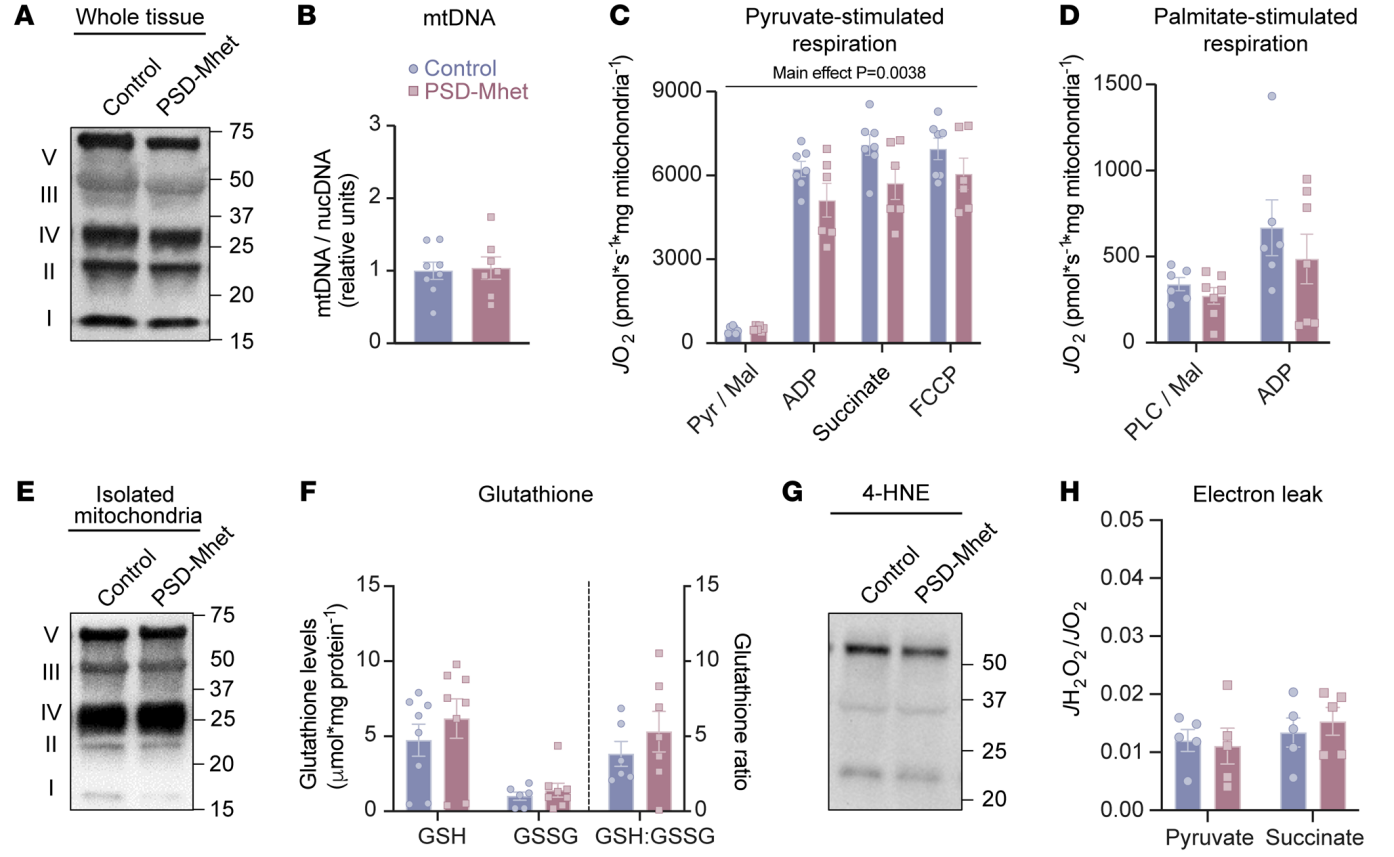
Reduction of mitochondrial pyruvate respiration by PSD haploinsufficiency is not mediated by oxidative stress. (**A**) Representative Western blot of respiratory protein complexes (I–V) of whole gastrocnemius muscle of SMC control and SMC PSD-Mhet (PSD muscle-specific heterozygous knockout) mice (*n* = 4–7 per group). (**B**) Nuclear to mitochondrial DNA in gastrocnemius muscles (*n* = 8 per group). (**C**) O_2_ utilization in isolated muscle mitochondria from gastrocnemius muscles with TCA cycle substrates using the same conditions described earlier (*n* = 6–7 per group). (**D**) O_2_ utilization in isolated muscle mitochondria from gastrocnemius muscles with fatty acid substrates using the same conditions described earlier (*n* = 6–7 per group). (**E**) Representative Western blot of respiratory complexes (I–V) of isolated muscle mitochondria from gastrocnemius muscles of SMC control and SMC PSD-Mhet mice (*n* = 5 per group). (**F**) Reduced (GSH) and oxidized (GSSG) glutathione levels in plantaris muscle (*n* = 8 per group). (**G**) Representative 4-hydroxynonenal (4-HNE) Western blot of whole muscle of SMC control and SMC PSD-Mhet mice (*n* = 6 per group). (**H**) Electron leak in isolated muscle mitochondria from gastrocnemius muscles stimulated with succinate or pyruvate (Pyr) and auranofin (*n* = 5 per group). PLC, palmitoyl-l-carnitine; ADP, adenosine diphosphate; FCCP, carbonyl cyanide-*p*-trifluoromethoxyphenylhydrazone. All data are from male control and PSD-Mhet mice. Data represent mean ± SEM. *P* values generated by 2-tailed, equal-variance, Student’s *t* test (**B**) or by 2-way ANOVA with Tukey’s post hoc test (**C**, **D**, **F**, and **H**).

Similar to our results with the SMC intervention in wild-type mice, PSD haploinsufficiency did not alter mitochondrial content in skeletal muscle (Figure 5, A and B, and Supplemental Figure 5A). High-resolution respirometry experiments revealed that low mitochondrial PE coincided with reduced pyruvate-stimulated *J*O_2_, without affecting OXPHOS protein content per unit of mitochondria (Figure 5, C–E, and Supplemental Figure 5B). Unlike homozygous deletion of PSD (15), heterozygous knockout of PSD did not promote oxidative stress or mitochondrial electron leak (Figure 5, F–H, and Supplemental Figure 5C). Taken together, these findings are consistent with the notion that low mitochondrial PE is sufficient to drive systemic and skeletal muscle metabolic inflexibility. In addition to physical inactivity, high-fat diet feeding is also known to induce metabolic flexibility (37). However, phenotypes for whole-body glucose metabolism or skeletal muscle mitochondrial function were not different between control and PSD-Mhet mice after high-fat diet feeding (Supplemental Figure 6, A–P). These observations suggest that reduction in mitochondrial PE influences metabolic flexibility by acting on pathways that are activated during physical inactivity but not with high-fat diet feeding. To delve deeper into the mechanism by which mitochondrial PE abundance facilitates pyruvate metabolism, we performed additional experiments in murine C2C12 myotubes.

### Mitochondrial PE deficiency impairs pyruvate metabolism.

**Figure 6:**
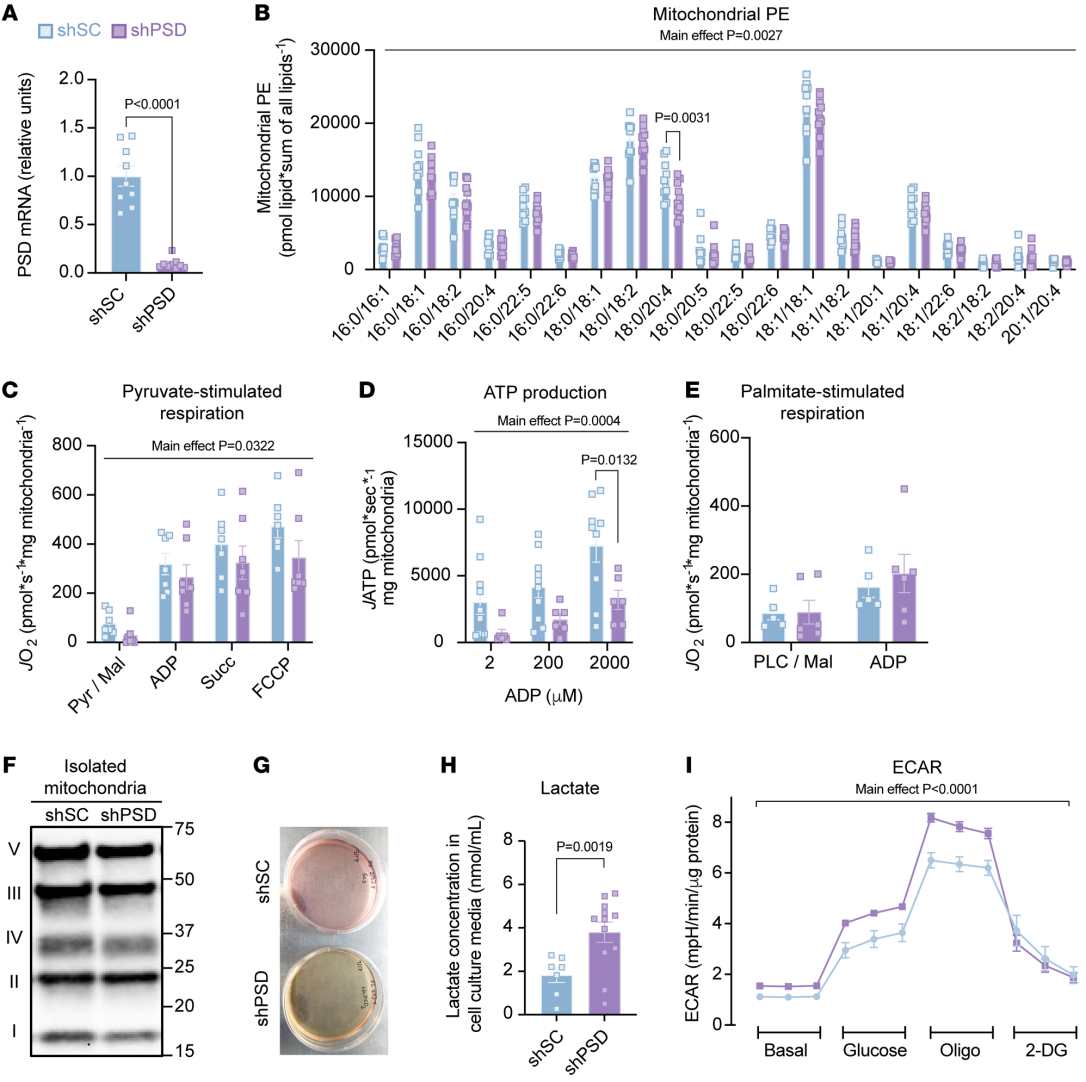
Mitochondrial PE deficiency impairs pyruvate metabolism. (**A**) PSD mRNA abundance in scrambled control (shSC) and PSD-knockdown (shPSD) C2C12 myotubes (*n* = 9 per group). (**B**) PE levels from isolated mitochondria of shSC and shPSD cells (*n* = 9–10 per group). (**C**) O_2_ consumption with Krebs cycle substrates using the same conditions described earlier (*n* = 7 per group). (**D**) ATP (adenosine triphosphate) production from isolated mitochondria of shSC and shPSD myotubes measured in the presence of 0.5 mM malate (Mal), 5 mM pyruvate (Pyr), 10 mM succinate (Succ), and either 2, 200, or 2,000 μM ADP (*n* = 7–10 per group). (**E**) O_2_ consumption with fatty acid substrates using the same conditions described earlier (*n* = 5–6 per group). (**F**) Representative Western blot of respiratory complexes I–V in isolated mitochondria from shSC and shPSD cells (*n* = 5–6 per group). (**G**) Representative image of media color from cell culture plates. (**H**) Quantification of lactate production in the media after 24 hours (*n* = 7–12 per group). (**I**) Seahorse extracellular acidification rate (ECAR) (*n* = 10 replicates per group). PLC, palmitoyl-l-carnitine; Oligo, oligomycin; 2-DG, 2-deoxyglucose. Data represent mean ± SEM. *P* values generated by 2-tailed, equal-variance, Student’s *t* test (**A** and **H**) or by 2-way ANOVA with Tukey’s post hoc test (**B**–**E** and **I**).

To study the effects of low mitochondrial PE, C2C12 myotubes were subjected to lentivirus-mediated knockdown with shRNA encoding either scrambled control (shSC) or PSD (shPSD), which was confirmed by quantitative real-time PCR (Figure 6A). We took advantage of the slow turnover rate for phospholipid molecules and performed all experiments 3 days after lentiviral infection to model modest reductions in some mitochondrial PE species (Figure 6B and Supplemental Figure 7A). Consistent with our observations in vivo, PSD knockdown attenuated pyruvate-stimulated *J*O_2_ or *J*ATP (Figure 6, C and D), but not palmitate-stimulated *J*O_2_ (Figure 6E). PSD knockdown also had no effect on OXPHOS content (total cellular or mitochondrial), mitochondrial electron leak, or oxidative stress (Figure 6F and Supplemental Figure 7, B–G). These findings indicate that cell-autonomous effects of PSD deletion are responsible for the phenotype observed in vivo.

Knockdown of PSD very strikingly accelerated the yellowing of the culture medium compared with shSC cells (Figure 6G). Yellowing of cell culture media is usually indicative of higher acidification rate due to lactate production (38). Indeed, lactate concentration in the medium was substantially elevated in shPSD cells compared with shSC controls (Figure 6H), and analysis of C2C12 myotubes on the Seahorse Bioanalyzer revealed increased extracellular acidification rate with PSD deletion (Figure 6I). Together, these data likely indicate that low PE causes mitochondria to become resistant to pyruvate metabolism (39, 40).

**Figure 7:**
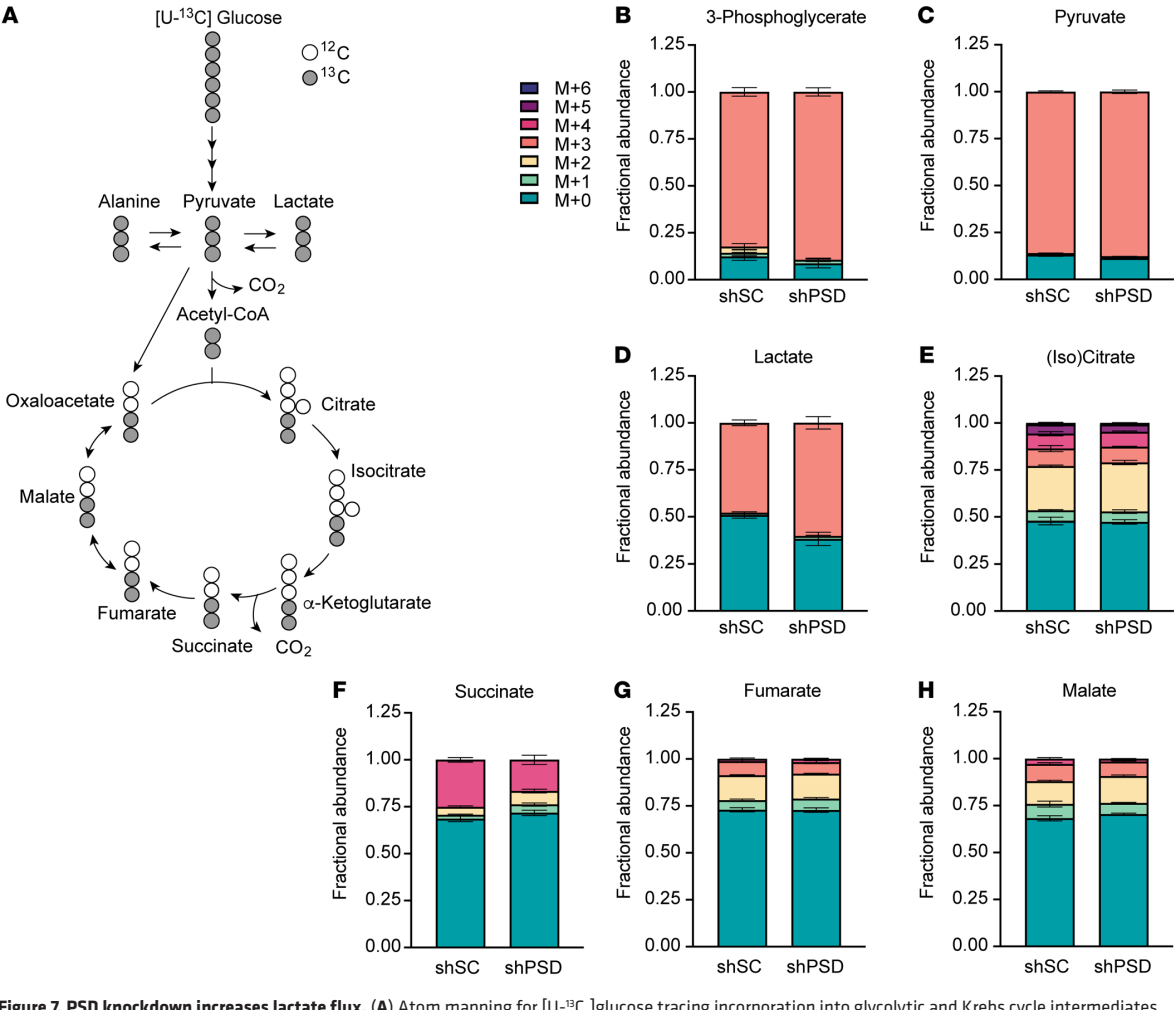
PSD knockdown increases lactate flux. (**A**) Atom mapping for [U-^13^C_6_]glucose tracing incorporation into glycolytic and Krebs cycle intermediates. White circles represent ^12^C atoms, while black circles signify ^13^C atoms. (**B**–**H**) Isotope labeling pattern between scrambled control (shSC) and PSD-knockdown (shPSD) myotubes for intracellular 3-phosphoglycerate (**B**), pyruvate (**C**), lactate (**D**), (iso)citrate (**E**), succinate (**F**), fumarate (**G**), and malate (**H**) (*n* = 4–5 per group). Data represent mean ± SEM.

**Figure 8:**
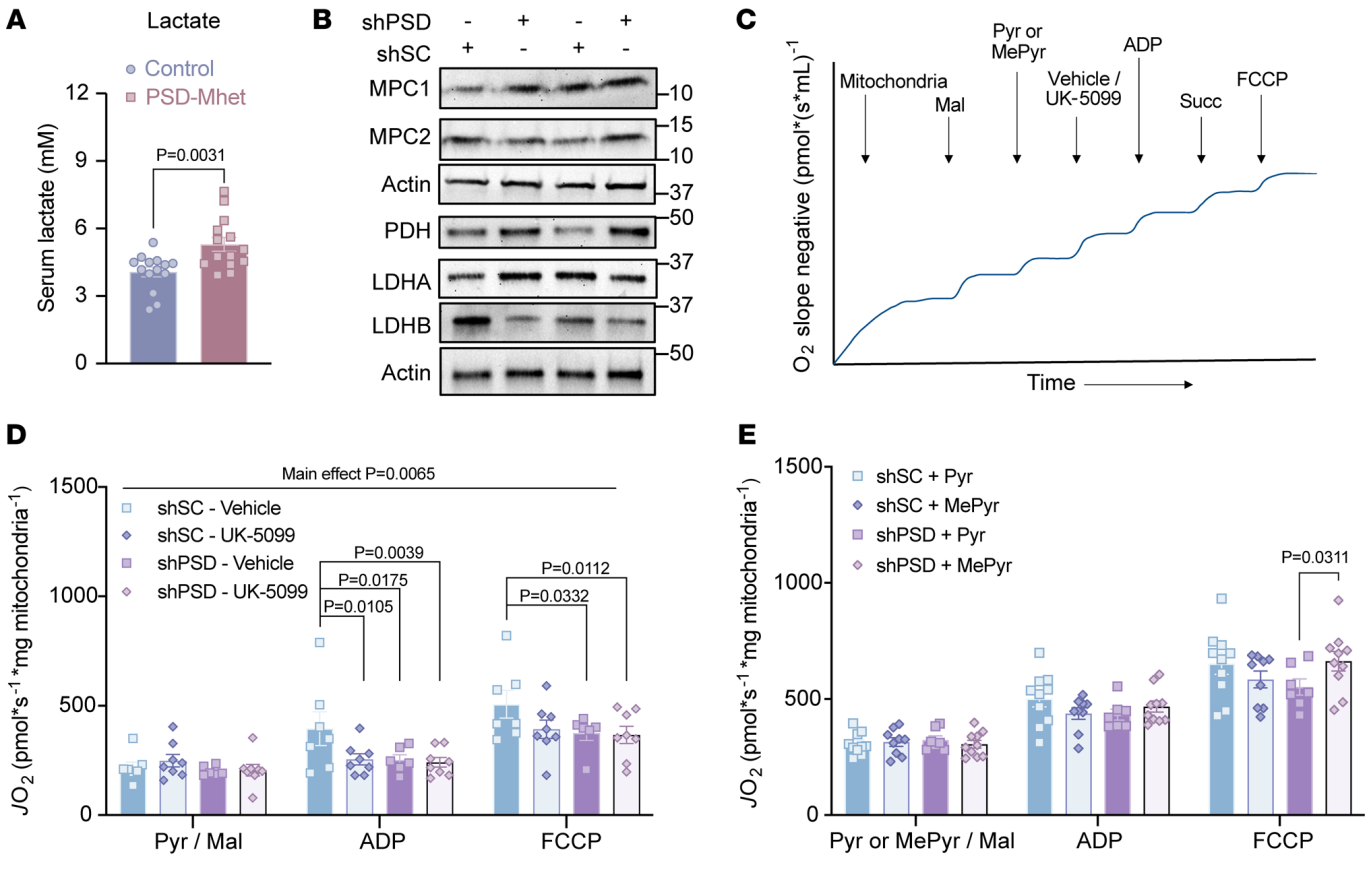
Mitochondrial PE facilitates pyruvate entry. (**A**) Circulating lactate levels from SMC control and PSD-Mhet mice (*n* = 14 per group). (**B**) Representative Western blot of MPC1, MPC2, PDH, LDHA, LDHB, and actin between scrambled control (shSC) and PSD-knockdown (shPSD) C2C12 myotubes (*n* = 6 per group). (**C**) Schematic of the order in which UK-5099 and MePyr were injected during high-resolution respirometry experiments. (**D**) Pyruvate-dependent O_2_ consumption in isolated mitochondria from shSC and shPSD myotubes in the presence or absence of the MPC inhibitor UK-5099 (100 nM) and the same Krebs cycle substrate conditions described above (*n* = 6–8 per group). (**E**) Pyruvate-dependent respiration in isolated mitochondria with Krebs cycle substrate conditions described above with either pyruvate or MePyr as a substrate (*n* = 6–10 per group). MPC1, mitochondrial pyruvate carrier complex 1; MPC2, mitochondrial pyruvate carrier complex 2; PDH, pyruvate dehydrogenase; LDHA, lactate dehydrogenase isoform A; LDHB, lactate dehydrogenase isoform B. Data represent mean ± SEM. *P* values generated by 2-tailed Student’s *t* test (**A**) or 2-way ANOVA with Tukey’s post hoc test (**D** and **E**).

To more closely examine intracellular pyruvate metabolism, we performed stable isotope tracing using uniformly labeled ^13^C-glucose (Figure 7, A–H, and Supplemental Figure 8). Targeted mass spectrometry analyses revealed that labeling for glycolytic metabolites leading up to pyruvate was elevated with PSD knockdown (Figure 7, B and C), suggesting that low mitochondrial PE does not compromise glucose-to-pyruvate metabolism. Consistent with increased lactate concentration in the medium, lactate labeling was higher in shPSD cells compared with shSC (Figure 7D). In contrast, low mitochondrial PE was not associated with increased labeling toward TCA intermediates (Figure 7, E–H), suggesting that flux toward lactate, and not TCA cycle, explains the increased labeling for the glycolytic metabolites. Similarly, circulating lactate was greater in SMC PSD-Mhet mice compared with SMC control mice (Figure 8A). These findings are consistent with the notion that mitochondrial PE deficiency impairs mitochondrial pyruvate metabolism.

### Mitochondrial PE facilitates mitochondrial pyruvate entry.

We sought to identify the mechanism by which low mitochondrial PE attenuates pyruvate metabolism. Surprisingly, PSD deletion did not reduce protein or mRNA abundance of mitochondrial pyruvate carriers (MPC1 and MPC2) or pyruvate dehydrogenase (PDH) (Figure 8B and Supplemental Figure 9, A–C), suggesting that attenuated pyruvate metabolism is not explained by changes in abundance of these proteins. In fact, there was a statistically significant increase in lactate dehydrogenase and a trend for an increase in PDH with PSD deletion. PSD is localized at the inner mitochondrial membrane (IMM) to generate PE. Thus, we reasoned that the mitochondrial PE may regulate the activity of MPC, which also resides in the IMM (41, 42).

To test this possibility, we took a 2-pronged approach to link MPC to a defect in pyruvate metabolism (Figure 8C). First, we performed pyruvate-stimulated respirometry with or without the MPC inhibitor UK-5099 (43). Consistent with UK-5099’s action on MPC, pyruvate-stimulated *J*O_2_ was significantly reduced in shSC myotubes (Figure 8D). As expected, MPC inhibition did not completely suppress *J*O_2_ owing to anaplerosis. Strikingly, MPC-inhibited *J*O_2_ in shSC cells was similar to *J*O_2_ in shPSD cells without UK-5099, consistent with the notion that reduced *J*O_2_ in shPSD cells is due to attenuated MPC activity. Furthermore, UK-5099 had no effect on *J*O_2_ in shPSD cells, confirming that residual *J*O_2_ in shPSD cells is independent of pyruvate entry via MPC. Second, we compared *J*O_2_ in response to pyruvate or methyl pyruvate (MePyr). MePyr is a pyruvate analog that can bypass the MPC, diffuse freely into the mitochondrial matrix, and subsequently be demethylated to become mitochondrial pyruvate (44). MePyr rescued *J*O_2_ in shPSD myotubes to pyruvate-stimulated *J*O_2_ levels in shSC cells (Figure 8E). Taken together, these findings suggest that low mitochondrial PE attenuates MPC activity to inhibit mitochondrial pyruvate metabolism.

## Discussion

Skeletal muscle disuse or physical inactivity is linked to 35 chronic diseases (4, 45). Many of these conditions are attributed to metabolic disturbances caused by sedentary behavior. Nevertheless, the mechanisms by which physical inactivity alters systemic and skeletal muscle metabolism have been poorly defined, likely because of the lack of preclinical models (11, 12). In this study, we developed a novel mouse model of inactivity that reliably induces metabolic inflexibility in male C57BL/6J mice. Metabolic inflexibility was likely driven by pyruvate resistance in skeletal muscle mitochondria. We implicate inactivity-induced downregulation of mitochondrial PE as a driver of pyruvate resistance. Skeletal muscle–specific deletion of PSD upon SMC insult in mice was sufficient to recapitulate metabolic inflexibility and mitochondrial pyruvate resistance in vivo and in vitro. Using stable isotope tracing and high-resolution respirometry, we demonstrate that PE likely directly acts on MPC to facilitate mitochondrial pyruvate entry.

Oxidative stress has been implicated in pathogenesis of inactivity-induced metabolic inflexibility (4, 45). Indeed, skeletal muscle oxidative stress is commonly manifested in many of the traditional models of mouse disuse (11, 12). However, while these models are useful in studying muscle atrophy, mice do not develop systemic and skeletal muscle metabolic adaptation observed with human sedentary behavior (11, 12). In our newly developed SMC model, metabolic inflexibility and suppression of glucose metabolism were similar to those in human bed rest studies, but muscles from this model of inactivity did not exhibit oxidative stress (glutathione, lipid hydroperoxides, mitochondrial electron leak). Notably, our findings from the SMC model reconcile with results from human bed rest studies that oxidative stress cannot explain metabolic inflexibility (36).

Previously we demonstrated that muscle mitochondrial PE becomes elevated with exercise training and decreased with hind-limb unloading (15). There are no studies that examined muscle mitochondrial PE in humans, but total cellular PE has been linked with insulin sensitivity (27). In subjects with type 2 diabetes, muscle PE content was lower in comparison with obese normosensitive individuals. We have previously demonstrated the role of PE generated by the Kennedy pathway (46, 47), where suppression of PE synthesis at the ER increased, not decreased, skeletal muscle insulin sensitivity with high-fat feeding. There is evidence that syntheses of PE at ER or mitochondria might become upregulated to compensate each other (46), which might contribute to phenotypes found in these papers. Together, these findings highlight the complex interactions between muscle glucose metabolism and subcellular lipid metabolism. They also reinforce the notion that the pools of PE generated at ER and those generated in mitochondria remain distinct.

Unlike oxidative stress, SMC reduced skeletal muscle mitochondrial PE concomitantly with the development of metabolic inflexibility. What are the mechanisms by which exercise or inactivity promotes changes in muscle mitochondrial PE? In our previous study, as well as in the current study, changes in mitochondrial PE coincided with mRNA abundance of PSD, an enzyme that generates PE in the inner mitochondrial membrane (IMM). We believe that changes in PSD levels likely drive the changes in mitochondrial PE abundance. It is currently unknown whether PSD activity is regulated by posttranslational modification. It is also possible that there are changes in the upstream mechanism for mitochondrial PE synthesis. PSD generates PE from mitochondrial PS, which is synthesized by PS synthase 1 and 2 in the ER (48, 49) and transported to mitochondria via Prelid3b (50). Finally, it would be important to determine the mechanism of the transcriptional control of PSD.

For an unknown reason, PE generated at the ER by the Kennedy pathway does not enter mitochondria (16). This is exemplified by findings that inhibition of PE synthesis at the ER does not reduce mitochondrial function in skeletal muscle (46, 47). In fact, deletion of ECT (CTP:phosphoethanolamine cytidylyltransferase, an intermediate step in PE synthesis) increases mitochondrial content, an observation that may be explained by a compensatory increase in muscle PSD (46). Similarly, deletion of CEPT1 (choline/ethanolamine phosphotransferase, the final step in PE synthesis) increases skeletal muscle glucose metabolism (47). Two caveats to our data on mitochondrial PE are worth noting. First, our lipidomic analyses were performed on mitochondrial preparations that are not exclusively IMM where MPC resides. They also contain outer mitochondrial membrane and other organelle contaminants that are also highly abundant in PE. While PSD almost exclusively contributes PE in IMM, PE in these contaminants is produced by the Kennedy pathway. Thus, it is very likely that our data on mitochondrial PE underestimate the true concentration of PE in IMM by a meaningful margin. A technological breakthrough to provide spatial resolution on IMM lipids is needed to better understand the exact nature of these changes. Second, it is worth noting that PE species distribution is quite different in muscles versus C2C12 myotubes. Acyl-chain combinations on PE are predicted to influence how these lipids influence IMM enzymes as well as membrane properties. The predominant PE species in vivo appear to contain the acyl chain 22:6 in the sn-2 position, a preferred substrate of PSD, whereas these species are much lower compared with other PE species in C2C12 myotubes. The differences are likely enabled by differences in fatty acid and/or ethanolamine availability between in vitro and in vivo (ZZ Oemer; https://doi.org/10.1016/j.celrep.2020.02.115). Overall, combined with our previous report on muscle-specific homozygous deletion of PSD (15), the current study emphasizes that the mitochondrial PE pool remains distinct from that of the ER. This is also consistent with findings in yeast, as PE generated by PSD with a forced localization at the outer mitochondrial membrane or ER has differential cellular consequences (51).

On a similar note, one of the critical findings of this study was that low mitochondrial PE coincided with pyruvate resistance, but not with palmitate-stimulated *J*O_2_. We demonstrate that PE likely directly facilitates MPC to promote mitochondrial pyruvate uptake, which takes place across the IMM where PE is enriched. Meanwhile, the rate-limiting step for fatty acid oxidation is at the step of carnitine palmitoyl transferase-1 (CPT1), which is localized on the outside of the outer mitochondrial membrane (52). Not only is CPT1 not a transmembrane protein, but it is also localized at the outer mitochondrial membrane where PE is less concentrated (53). The enzyme equivalent to the MPC for fatty acid oxidation is carnitine/acylcarnitine translocase, which is located in the IMM, but this enzyme is not the rate-limiting step of palmitate entry nor palmitate oxidation (54, 55). Thus, we believe that differential subcellular localization of the rate-limiting step for pyruvate or palmitate oxidation contributes to the disproportionate influence of low mitochondrial PE on substrate preference.

Yellowing of cell culture medium was the most apparent and robust phenotype observed with PSD knockdown in vitro. Our flux experiments reveal that this is a direct result of accelerated flux of glucose toward lactate. Experiments with UK-5099 and MePyr suggest that pyruvate resistance in PSD-deficient cells is attributable to the effects of PE on MPC. Multiple studies demonstrate that inhibiting MPC results in impairments to oxidization of glycolytic substrates (41, 42, 56, 57). We believe that the effects of PE deficiency on MPC represent the mechanism underlying the metabolically inflexible phenotype observed in PSD-Mhet mice. We further reason that metabolic inflexibility caused by sedentariness is attributable to low mitochondrial PE, which in turn reduces mitochondrial pyruvate entry. It would be important for future studies to elucidate whether PE directly affects the stability of MPC or its posttranslational modifications to regulate pyruvate entry.

Lactate infusion is known to rapidly suppress insulin-stimulated glycolysis and intracellular glucose metabolism that leads to a decrease in glucose uptake (58). Likewise, we speculate that low mitochondrial PE attenuates pyruvate import and oxidation, suppressing intracellular glycolytic flux to reduce glucose uptake. Nonetheless, the effects of physical inactivity on whole-body and skeletal muscle metabolism are pleiotropic, and we cannot effectively rule out the contribution of alternate mechanisms that underlie reduced glucose transport with SMC.

In conclusion, the current study demonstrates a mechanism by which PE facilitates mitochondrial pyruvate entry. We show that a modest reduction in mitochondrial PE is sufficient to promote resistance toward pyruvate oxidation both in vitro and in vivo. These observations were further extrapolated by findings that pyruvate resistance can be rescued by the membrane-permeable MePyr, and that the MPC inhibitor UK-5099 can phenocopy the effects of low mitochondrial PE. We propose that this process drives the metabolic inflexibility induced by physical inactivity in male mice. Resistance to pyruvate oxidation may represent a selective advantage for mammals in a state of reduced energy demand, such that substrates are shunted away from skeletal muscle and stored away for subsequent energetic needs. In the modern age of abundant food supply, inactivity-driven resistance to glycolytic substrates can exacerbate the development of metabolic diseases.

### Limitations.

The physiological responses that occur as a result of physical inactivity are highly complex and appear to be sex dependent. The majority of human bed rest or reduced-activity studies have largely been conducted in males (7, 27, 36, 59), while inactivity studies in females have focused on postmenopausal women (60, 61). In our study, female mice were resistant to SMC-induced metabolic inflexibility. The cause of this difference is unclear, but our findings suggest that muscle mitochondria from male mice contained higher PE in comparison with female mice in sham condition, such that they were more prone to inactivity-induced reduction in mitochondrial PE and suppression of mitochondrial pyruvate metabolism. It is unclear whether such sexually dimorphic response persists in humans. We believe that more studies examining both sexes, in humans and in mice, are needed to study the influence of sedentary behavior on metabolic homeostasis.

## Methods

### Sex as a biological variable.

Both male and female mice were examined. Differences between sexes were extrapolated to study mechanisms.

### Animals.

Eight-week-old C57BL/6J mice were purchased from The Jackson Laboratory (strain 000664) for initial small mouse cage (SMC) experiments. Heterozygous PSD-Mhet mice were generated by crossing of our conditional PSD knockout (PSDcKO^+/+^) mice (previously described; ref. 15). PSDcKO^+/+^ mice harbor *loxP* sites flanking exons 4–8 of the mouse PSD gene. These mice were crossed with HSA-MerCreMer mice (HSA-MerCreMer, tamoxifen-inducible α-human skeletal actin Cre, courtesy of K. Esser, University of Florida, Gainesville, Florida, USA). All mice were bred onto C57BL/6J background and were born at normal Mendelian ratios. Tamoxifen (final concentration of 10 mg/mL) was injected intraperitoneally (7.5 μL/g of body weight) to PSD-Mhet mice and their respective controls for 5 consecutive days. After a 2-week washout, mice were studied as sham, SMC (discussed further below), or high-fat diet feeding (Western diet, TD.88137, Envigo) groups. It is noteworthy that because tamoxifen is an estrogen receptor antagonist that may influence metabolism, data from mice that were injected with tamoxifen (control and PSD-Mhet mice) may not always be directly comparable to data from mice that were not (wild-type mice with or without SMC). Differences in housing facility, age, and dates of experiments also contribute to these differences. Mice were maintained on a 12-hour light/12-hour dark cycle in a temperature-controlled room at 22°C. All animals were fasted for 4 hours before tissue collection or experiments. Before all terminal experiments and tissue harvesting, mice were given an intraperitoneal injection of 80 mg/kg ketamine and 10 mg/kg xylazine. All protocols were approved by the Institutional Animal Care and Use Committee at the University of Utah.

### Small mouse cage.

Modified and further developed from Mahmassani et al. (14) and Marmonti et al. (13), SMC is a rectangular box produced from acrylic plastic, made at the University of Utah’s Machine Shop Core. Bedding is placed one-third of the height leaving 4 cm of clearance height. Air holes are designed on all 4 sides to facilitate air circulation. One air hole on the side was plugged with a Hydropac water lixit (Lab Products Inc.) providing water ad libitum, and one air hole on the top is compatible with the hydration system of the Columbus Instruments Oxymax Lab Animal Monitoring System (CLAMS) for determination of whole-animal energy expenditure. Abundance of food is provided on top of the bedding to allow ad libitum food consumption. Variable water leakage and crumbling of food are caveats to the attainment of accurate food and water intake in the SMC. Bedding, food, and water were changed every 2–3 days to ensure cleanliness. Two SMC cages can fit in 1 regular mouse cage. Some experiments were performed with sham and SMC mice housed in pairs, while other experiments were performed with separate cages for sham and SMC mice.

### Indirect calorimetry.

The Columbus Instruments Lab Monitoring System was used to measure VO_2_, respiratory exchange ratio (RER; VCO_2_/VO_2_), food intake, and physical activity (for sham animals only) of sham and SMC mice during week 7 or 8 of SMC. Mice were individually caged and acclimated for over 24 hours in the system before data were collected. Body composition was determined using the Bruker Minispec NMR.

### Glucose tolerance test.

Intraperitoneal glucose tolerance tests were performed by injection of 1 mg glucose per gram body mass during week 8 of SMC, at least 3 days before sacrifice. Mice were fasted for 4 hours before glucose injection. Blood glucose was measured before glucose injection and 15, 30, 60, and 120 minutes after injection via tail bleed with a handheld glucometer (Bayer Contour 7151H). In a separate set of experiments, mice were injected with 1 mg glucose per gram body mass, and blood was taken from the facial vein at the 30-minute time point for insulin quantification.

### Ex vivo skeletal muscle [^3^H]2-deoxy-d-glucose uptake.

Ex vivo glucose uptake was measured in soleus muscle as previously described (47). In brief, soleus muscles were dissected and placed in a recovery buffer (Krebs-Henseleit buffer [KHB] with 0.1% BSA, 8 mM glucose, and 2 mM mannitol) at 37°C for 10 minutes. After incubation in recovery buffer, muscles were moved to preincubation buffer (KHB with 0.1% BSA, 2 mM sodium pyruvate, and 6 mM mannitol) with or without 200 μU/mL insulin for 15 minutes for soleus and with or without 600 μU/mL insulin for extensor digitorum longus. After preincubation, muscles were placed in incubation buffer (KHB with 0.1% BSA, 9 mM [^14^C]mannitol, 1 mM [^3^H]2-deoxyglucose) with or without 200 μU/mL insulin for 15 minutes. Contralateral muscles were used for basal or insulin-stimulated measurements. After incubation, muscles were blotted dry on ice-cold filter paper, snap-frozen, and stored at –80°C until analyzed with liquid scintillation counting.

### Serum insulin, glucose, and cortisol quantification.

Blood was collected from facial vein either before anesthesia or at the 30-minute time point of the glucose tolerance test. Blood was then placed at room temperature for 20 minutes to clot before centrifugation at 2,000*g* for 10 minutes at 4°C. The supernatant (serum) was collected, placed in a new tube, and stored at –80°C until analysis. Serum insulin levels were quantified using a Mouse Insulin ELISA kit (catalog 90080, Crystal Chem). Serum glucose was quantified using a Mouse Glucose Assay Kit (catalog 81692, Crystal Chem). Serum cortisol levels were quantified by the DetectX ELISA kit (catalog K003-H1W, Arbor Assays).

### High-resolution respirometry and fluorometry.

Mitochondrial O_2_ utilization was measured using the Oroboros O2K Oxygraphs. Skeletal muscle tissues were minced in mitochondria isolation medium (300 mM sucrose, 10 mM HEPES, 1 mM EGTA) and subsequently homogenized using a Teflon-glass system. Homogenates were then centrifuged at 800*g* for 10 minutes, after which the supernatant was taken and centrifuged at 12,000*g* for 10 minutes. The resulting pellet was carefully resuspended in mitochondria isolation medium. Isolated mitochondria were then added to the oxygraphy chambers containing assay buffer (MES potassium salt 105 mM, KCl 30 mM, KH_2_PO_4_ 10 mM, MgCl_2_ 5 mM, BSA 0.5 mg/mL). Respiration was measured in response to the following substrates: 0.5 mM malate, 5 mM pyruvate, 5 mM glutamate, 10 mM succinate, 1.5 μM FCCP, 0.02 mM palmitoylcarnitine, 5 mM l-carnitine. ATP production was measured fluorometrically using a Horiba Fluoromax 4 (Horiba Scientific), by enzymatic coupling of ATP production to NADPH synthesis as previously described (62). Respiration and ATP production were measured in the presence of 2 mM ADP, unless otherwise stated.

For inhibitor experiments in mitochondria isolated from shSC and shPSD myotubes, the mitochondrial pyruvate carrier (MPC) inhibitor UK-5099 (5048170001, Sigma-Aldrich) was used to inhibit MPC activity. To induce a submaximal drop of pyruvate-dependent respiration, 100 nM UK-5099 was used at a submaximal concentration and injected into the oxygraph chamber after the addition of malate and pyruvate. Respiration was measured in response to the following substrates: 0.5 mM malate, 5 mM pyruvate, 2 mM ADP, and 1 μM FCCP. To evaluate whether pyruvate-dependent respiration was compromised in shSC and shPSD mitochondria, respiration was measured in response to either 5 mM pyruvate or 5 mM methyl pyruvate (371173, Sigma-Aldrich) along with the above substrates.

### H_2_O_2_ measurements.

Mitochondrial H_2_O_2_ emission was determined in isolated mitochondria from skeletal muscle. All *J*H_2_O_2_ experiments were performed in buffer Z supplemented with 10 mM Amplex UltraRed (Invitrogen), 20 U/mL CuZn superoxide dismutase, and 25 mM blebbistatin (for permeabilized muscle fibers only). Briefly, isolated mitochondria or permeabilized fibers were added to 1 mL of assay buffer containing Amplex UltraRed, which produces a fluorescent product when oxidized by H_2_O_2_. H_2_O_2_ emission was measured after the addition of 10 mM succinate or 5 mM pyruvate for a final concentration. The appearance of the fluorescent product was measured by a Horiba Fluoromax 4 fluorometer (excitation/emission = 565 nm/600 nm).

### Seahorse assay.

Extracellular acidification rate (ECAR) was measured in C2C12 myoblasts using a Seahorse XF96 Analyzer. Myoblasts were plated at 5 × 10 cells per well and grown in lentiviral medium for 48 hours. C2C12 cells were selected with puromycin throughout differentiation for 3 days. The real-time ECAR was measured using the XFe96 extracellular flux analyzer with the Glycolysis Stress Kit (Agilent Technologies) following the manufacturer’s instructions. The measurement was normalized to total protein determined by Pierce BCA Protein Assay Kit (Thermo Fisher Scientific). Briefly, cells were seeded on XF96 cell culture microplates (Seahorse Bioscience) at a seeding density of 5.0 × 10^3^ cells per well. Before assay, cells were rinsed twice and kept in pre-warmed XF basic assay medium (pH 7.4) supplemented with 2 mM glutamine in a 37°C non-CO_2_ incubator for an hour. Then the rate was measured at 37°C in 14 replicates (separate wells) by use of the following compounds in succession: 10 mM glucose, 1 μM oligomycin, and 50 mM 2-deoxyglucose. Basal ECAR was measured before drug exposure. The glycolytic function metrics were calculated by Seahorse Wave Desktop Software as directed in the glycolysis stress kit manual (Agilent Technologies).

### Glutathione.

Skeletal muscle reduced glutathione (GSH) and oxidized glutathione (GSSG) were measured using the fluorometric GSH/GSSG Ratio Detection Assay Kit II (Abcam 205811). Briefly, whole plantaris muscle was homogenized in lysis buffer containing 0.1% SDS, 0.1% sodium deoxycholate, 1% Triton X-100, 50 mM Tris-HCl (pH 7.6), 5 mM EDTA, 150 mM NaCl, and protease and phosphatase inhibitor cocktail, deproteinized using the Deproteinizing Sample Kit – TCA (Abcam 204708), nutated at 4°C for 1 hour, and centrifuged at 4°C for 15 minutes at 12,000*g*. The supernatant was collected, and protein concentrations were determined using the Pierce BCA Protein Assay (Thermo Fisher Scientific). Supernatant was then used to determine GSH and total glutathione. Fluorescence was measured at excitation/emission = 490/520 nm with a fluorescence microplate reader.

### Enzyme activity assays.

pyruvate dehydrogenase (Ab109902), phosphofructokinase (Ab155898), and citrate synthase (Ab119692) activity assays were performed using activity assay kits from Abcam.

### Cell culture.

C2C12 myoblasts (ATCC CRL-1772) were grown in high-glucose DMEM (4.5 g/L glucose with l-glutamine; Gibco 11965-092) supplemented with 10% FBS (heat-inactivated, certified, US origin; Gibco 10082-147), and 0.1% penicillin-streptomycin (10,000 U/mL; Gibco 15140122). C2C12 cells were differentiated into myotubes with low-glucose DMEM (1 g/L glucose with 4 mM l-glutamine and 110 mg/L sodium pyruvate; Gibco 11885-084) supplemented with 2% horse serum (defined; VWR 16777), and 0.1% penicillin-streptomycin.

### Lentivirus-mediated knockdown of PSD.

PSD expression was decreased using a pLKO.1 lentiviral-RNAi system. Plasmids encoding shRNA for mouse PISD (shPSD: TRCN0000115415) were obtained from MilliporeSigma. Packaging vector psPAX2 (ID 12260), envelope vector pMD2.G (ID 12259), and scrambled shRNA plasmid (SC: ID 1864) were obtained from Addgene. HEK293T cells in 10 cm dishes were transfected using 50 μL 0.1% polyethylenimine, 200 μL 0.15 M sodium chloride, and 500 μL Opti-MEM (with HEPES, 2.4 g/L sodium bicarbonate, and l-glutamine; Gibco 31985) with 2.66 μg of psPAX2, 0.75 μg of pMD2.G, and 3 μg of either scrambled or PISD shRNA plasmid. Cells were selected with puromycin throughout differentiation to ensure that only cells infected with shRNA vectors were viable.

### U-^13^C-glucose labeling in cultured myotubes.

For metabolite extraction, cold 90% methanol (MeOH) solution was added to each sample to give a final concentration of 80% MeOH to each cell pellet. Samples were then incubated at –20°C for 1 hour. After incubation, samples were centrifuged at 20,000*g* for 10 minutes at 4°C. The supernatant was then transferred from each sample tube into a labeled, fresh microcentrifuge tube. Process blanks were made using only extraction solvent and no cell culture. The samples were then dried in vacuo.

All gas chromatography–mass spectrometry (GC-MS) analysis was performed with an Agilent 5977b HES fit with an Agilent 7693A automatic liquid sampler. Dried samples were suspended in 40 μL of 40 mg/mL *O*-methoxylamine hydrochloride (MP Bio 155405) in dry pyridine (EMD Millipore PX2012-7) and incubated for 1 hour at 37°C in a sand bath. Twenty-five microliters of this solution was added to autosampler vials. Sixty microliters of *N*-methyl-*N*-trimethylsilyltrifluoracetamide (MSTFA; with 1% TMCS; Thermo Fisher Scientific TS48913) was added automatically via the autosampler and incubated for 30 minutes at 37°C. After incubation, samples were vortexed, and 1 μL of the prepared sample was injected into the gas chromatograph inlet in split mode with the inlet temperature held at 250°C. A 10:1 split ratio was used for analysis for the majority of metabolites. Any metabolites that saturated the instrument at the 10:1 split were analyzed at a 50:1 split ratio. The gas chromatograph had an initial temperature of 60°C for 1 minute followed by a 10°C/min ramp to 325°C and a hold time of 10 minutes. A 30-meter Agilent Zorbax DB-5MS with 10-meter Duraguard capillary column was used for chromatographic separation. Helium was used as the carrier gas at a rate of 1 mL/min.

Data were collected using Agilent MassHunter software. Metabolites were identified and their peak area was recorded using MassHunter Quant. Metabolite identity was established using a combination of an in-house metabolite library developed using pure purchased standards and the National Institute of Standards and Technology (NIST) and Fiehn libraries. There are a few reasons why a specific metabolite may not be observable through GC-MS. The metabolite may not be amenable to GC-MS because of its size, or a quaternary amine such as carnitine, or simply because it does not ionize well.

### Lipid extraction.

Liquid chromatography–mass spectrometry–grade (LC-MS–grade) solvents and mobile phase modifiers were obtained from Honeywell Burdick & Jackson (acetonitrile, isopropanol, formic acid), Fisher Scientific (methyl *tert*-butyl ether), and Sigma-Aldrich/Fluka (ammonium formate, ammonium acetate). Lipid standards were obtained from Avanti Polar Lipids. Lipids were extracted from mitochondria with a modified Matyash lipid extraction (63) using a biphasic solvent system of cold methanol, methyl *tert-*butyl ether (MTBE), and water. Briefly, a mixture of cold MTBE, methanol, and internal standards (Mouse SPLASH LIPIDOMIX, Avanti Polar Lipids 330707; and Cardiolipin Mix I, Avanti Polar Lipids LM6003) was added to mitochondria isolated from C2C12 myotubes or gastrocnemius skeletal muscle. Samples were sonicated for 60 seconds, then incubated on ice with occasional vortexing for 1 hour. After addition of 188 μL of PBS, the mixture was incubated on ice for 15 minutes and centrifuged at 12,000*g* for 10 minutes at 4°C. The organic (upper) layer was collected, and the aqueous layer was re-extracted with 1 mL of 10:3:2.5 (vol/vol/vol) MTBE/MeOH/water. The MTBE layers were combined for untargeted lipidomic analysis and dried under vacuum. The aqueous layer was centrifuged at 12,000*g* for 10 minutes at 4°C and dried under vacuum. Lipid extracts were reconstituted in 500 μL of 8:2:2 (vol/vol/vol) isopropanol (IPA)/acetonitrile (ACN)/water containing 10 mM ammonium formate and 0.1% formic acid. Concurrently, a process blank sample was prepared, and then a pooled quality control (QC) sample was prepared by taking equal volumes (~50 μL) from each sample after final resuspension.

### LC-MS analysis and data processing.

Lipid extracts were separated on an Acquity UPLC CSH C18 column (2.1 × 100 mm; 1.7 μm) coupled to an Acquity UPLC CSH C18 VanGuard precolumn (5 × 2.1 mm; 1.7 μm) maintained at 65°C connected to an Agilent HiP 1290 Sampler, Agilent 1290 Infinity pump, and Agilent 6545 Accurate Mass Q-TOF dual AJS-ESI mass spectrometer (Agilent Technologies). Samples were analyzed in a randomized order in both positive and negative ionization modes in separate experiments acquiring with the scan range *m*/*z* 100–1,700. Mobile phase A consisted of ACN/H_2_O (60:40, vol/vol) in 10 mM ammonium formate and 0.1% formic acid, and mobile phase B consisted of IPA/ACN/H_2_O (90:9:1, vol/vol/vol) in 10 mM ammonium formate and 0.1% formic acid. For negative-mode analysis the modifiers were changed to 10 mM ammonium acetate. The chromatography gradient for both positive and negative modes started at 15% mobile phase B, then increased to 30% B over 2.4 minutes; it then increased to 48% B from 2.4 to 3.0 minutes, then to 82% B from 3 to 13.2 minutes, then to 99% B from 13.2 to 13.8 minutes, where it was held until 16.7 minutes and then returned to the initial conditions and equilibrated for 5 minutes. Flow was 0.4 mL/min throughout, with injection volumes of 2 μL for positive and 10 μL for negative mode. Tandem mass spectrometry (MS/MS) was conducted using iterative exclusion, the same LC gradient at collision energies of 20 V and 27.5 V in positive and negative modes, respectively. For data processing, Agilent MassHunter (MH) Workstation and software packages MH Qualitative and MH Quantitative were used. The pooled QC (*n* = 8) and process blank (*n* = 4) were injected throughout the sample queue to ensure the reliability of acquired lipidomics data. For lipid annotation, accurate mass and MS/MS matching was used with the Agilent Lipid Annotator library and LipidMatch (64). Results from the positive and negative ionization modes from Lipid Annotator were merged based on the class of lipid identified. Data exported from MH Quantitative were evaluated using Excel, where initial lipid targets were parsed based on the following criteria. Only lipids with relative standard deviations less than 30% in QC samples were used for data analysis. Additionally, only lipids with background AUC counts in process blanks that were less than 30% of QC were used for data analysis. The parsed Excel data tables were normalized based on the ratio to class-specific internal standards, then to tissue mass and sum before statistical analysis.

### Supplemental Methods.

Further information can be found in Supplemental Methods.

### Statistics.

All data presented herein are expressed as mean ± SEM. The level of significance was set at *P* less than 0.05. Student’s *t* tests (2-tailed) were used to determine the significance between experimental groups, and 2-way ANOVA followed by Tukey’s honestly significant difference post hoc test was used where appropriate. The sample size (*n*) for each experiment is shown in the figure legends and corresponds to the sample derived from the individual mice or, for cell culture experiments, to an individual batch of cells. Unless otherwise stated, statistical analyses were performed using GraphPad Prism software.

### Study approval.

Experiments on animals were performed in strict accordance with the *Guide for the Care and Use of Laboratory Animals* of the NIH (National Academies Press, 2011). All animals were handled according to approved University of Utah Institutional Animal Care and Use Committee protocols (20-07007). The protocols were approved by the Committee on the Ethics of Animal Experiments of the University of Utah.

### Data availability.

Data are available in the Supporting Data Values file. RNA sequencing data sets were deposited in the NCBI’s Gene Expression Omnibus database (GEO GSE260612).

## Author contributions

PS contributed to conceptualization, data curation, formal analysis, validation, investigation, visualization, methodology, writing of the original draft, review and editing, and funding acquisition. GC contributed to conceptualization, investigation, data curation, and methodology. AAC contributed to conceptualization, data curation, formal analysis, and methodology. JAM and QP contributed to data curation, formal analysis, and resources. MJB, MYJ, HE, PJF, and PCO contributed to data curation. ZSM contributed to conceptualization and methodology. ADP, SW, and MAW contributed to data curation. EBT contributed to methodology. JEC contributed to methodology, resources, and supervision. MJD contributed to conceptualization, methodology, resources, and supervision. JR contributed to conceptualization and resources. KF contributed to conceptualization, formal analysis, validation, visualization, supervision, review and editing, resources, funding acquisition, and project administration.

## Supplementary Material

Supplemental data

Unedited blot and gel images

Supporting data values

## Version 1 04/23/2024

In-Press Preview

## Version 2 06/03/2024

Electronic publication
