# Supplementary material for: Sedentary behavior in mice induces metabolic inflexibility by suppressing skeletal muscle pyruvate metabolism
Source: J Clin Invest. 2024 Apr 23;134(11):e167371. doi: 10.1172/JCI167371 (PMC11142742; doi:10.1172/JCI167371)
Supplement: Supplemental data [file jci-134-167371-s244.pdf]

SUPPLEMENTAL MATERIALS

Sedentary behavior in mice induces metabolic inflexibility by suppressing skeletal muscle pyruvate metabolism

Piyarat Siripoksup,[1,2] Guoshen Cao,[1,3] Ahmad A. Cluntun,[1,3] J. Alan Maschek,[4,5] Quentinn Pearce,[4] Marisa J. Lang,[1,5] Mi-Young Jeong,[1,3] Hiroaki Eshima,[1,7] Patrick J. Ferrara,[1,5] Precious C. Opurum,[1,5] Ziad S. Mahmassani,[1,2,7] Alek D. Peterlin,[1,5] Shinya Watanabe,[1,5] Maureen A. Walsh,[1,2] Eric B. Taylor,[6] James E. Cox,[1,3,4] Micah J. Drummond,[1,2,7] Jared Rutter,[1,3,8] Katsuhiko Funai.[1,2,5,7,*]

[1]Diabetes & Metabolism Research Center, University of Utah, Salt Lake City, Utah, USA.

[2]Department of Physical Therapy & Athletic Training, University of Utah, Salt Lake City, Utah, USA.

[3]Department of Biochemistry University of Utah, Salt Lake City, Utah, USA.

[4]Metabolomics Core Research Facility, University of Utah, Salt Lake City, Utah, USA.

[5]Department of Nutrition & Integrative Physiology, University of Utah, Salt Lake City, Utah, USA.

[6]Fraternal Order of Eagles Diabetes Research Center, University of Iowa, Iowa City, Iowa, USA.

[7]Molecular Medicine Program, University of Utah, Salt Lake City, Utah, USA.

[8]Howard Hughes Medical Institute, University of Utah, Salt Lake City, Utah, USA.

*Correspondence:

Katsuhiko Funai, Ph.D.

Diabetes & Metabolism Research Center

University of Utah

15 N, 2030 E, Salt Lake City, UT 84112

Phone: (801) 585-1781

Fax: (801) 585-0701

Email: kfunai@health.utah.edu

Supplemental Methods

Western blotting

Tissues or cells were homogenized in lysis buffer containing 0.1% SDS, 0.1% sodium deoxycholate, 1% Triton X-100, 50 mM Tris-HCl pH 7.6, 5 mM EDTA, 150 mM NaCl, and protease and phosphatase inhibitor cocktail, nutated at 4°C for 1 hour, and centrifuged at 4°C for 15 min at 12,000*g*, and the supernatant was transferred to a new tube. Western blotting was performed as previously described (1), and samples were analyzed for protein abundance of OXPHOS (ab110413, Abcam), 4-HNE (ab46545, Abcam), MPC1 (generated by Jared Rutter), MPC2 (generated by Jared Rutter), PDH (3205S, Cell Signaling), LDHA (3582S, Cell Signaling), LDHB (sc-100775, SantaCruz Biotech), and Actin (A2066, Sigma-Aldrich).

RNA quantification

For quantitative polymerase chain reaction (qPCR) experiments, mouse tissues or cells were lysed in the TRIzol reagent (Thermo Fisher Scientific), and RNA was isolated using standard techniques. The iScript cDNA Synthesis Kit was used to reverse transcribe total RNA, and qPCR was performed with SYBR Green reagents (Thermo Fisher Scientific). Pre-validated primer sequences were obtained from mouse primer depot (https://mouseprimerdepot.nci.nih.gov/). All mRNA levels were normalized to RPL32 and primer sequences are provided (Table S1). For RNA sequencing, gastrocnemius muscle RNA from Sham and SMC mice were isolated with the Direct-zol RNA Miniprep Plus kit (Zymo Cat#: R2070). RNA library construction and sequencing were performed by the High-Throughput Genomics Core at the Huntsman Cancer Institute, University of Utah. RNA libraries were constructed using the NEBNext Ultra II Directional RNA Library Prep with rRNA Depletion Kit (human, mouse rat) and the following adapter reads: Read 1: AGATCGGAAGAGCACACGTCTGAACTCCAGTCA and Read 2:

AGATCGGAAGAGCGTCGTGTAGGGAAAGAGTGT. Sequencing was performed using the NovaSeq S4 Reagent Kit v1.5 150x150 bp Sequencing with >25 million reads per sample. Pathway analyses were performed by the Bioinformatics Core at the Huntsman Cancer Institute, University of Utah using the KEGG (Kyoto Encyclopedia of Genes and Genomes) Pathway Database. For differentially expressed genes, only transcripts with Padj < 0.05 and baseMean > 100 are included.

DNA isolation and quantitative PCR

Genomic DNA for assessments of mitochondrial DNA (mtDNA) was isolated using a commercially available kit according to the manufacturer's instructions (69504, Qiagen). Genomic DNA was added to a mixture of SYBR Green (Thermo Fisher Scientific) and primers. Sample mixtures were pipetted onto a 3840well plate and analyzed with QuantStudio 12K Flex (Life Technologies). The following primers were used: mtDNA fwd, TTAAGA-CAC-CTT-GCC-TAG-CCACAC; mtDNA rev, CGG-TGG-CTG-GCA-CGA-AAT-T; nucDNA fwd, ATGACG-ATA-TCG-CTG-CGC-TG; nucDNA rev, TCA-CTT-ACC-TGGTGCCTA-GGG-C.

References

1. Eshima H, Siripoksup P, Mahmassani ZS, Johnson JM, Ferrara PJ, Verkerke ARP, et al. Neutralizing mitochondrial ROS does not rescue muscle atrophy induced by hindlimb unloading in female mice. J Appl Physiol (1985). 2020;129(1):124-32.

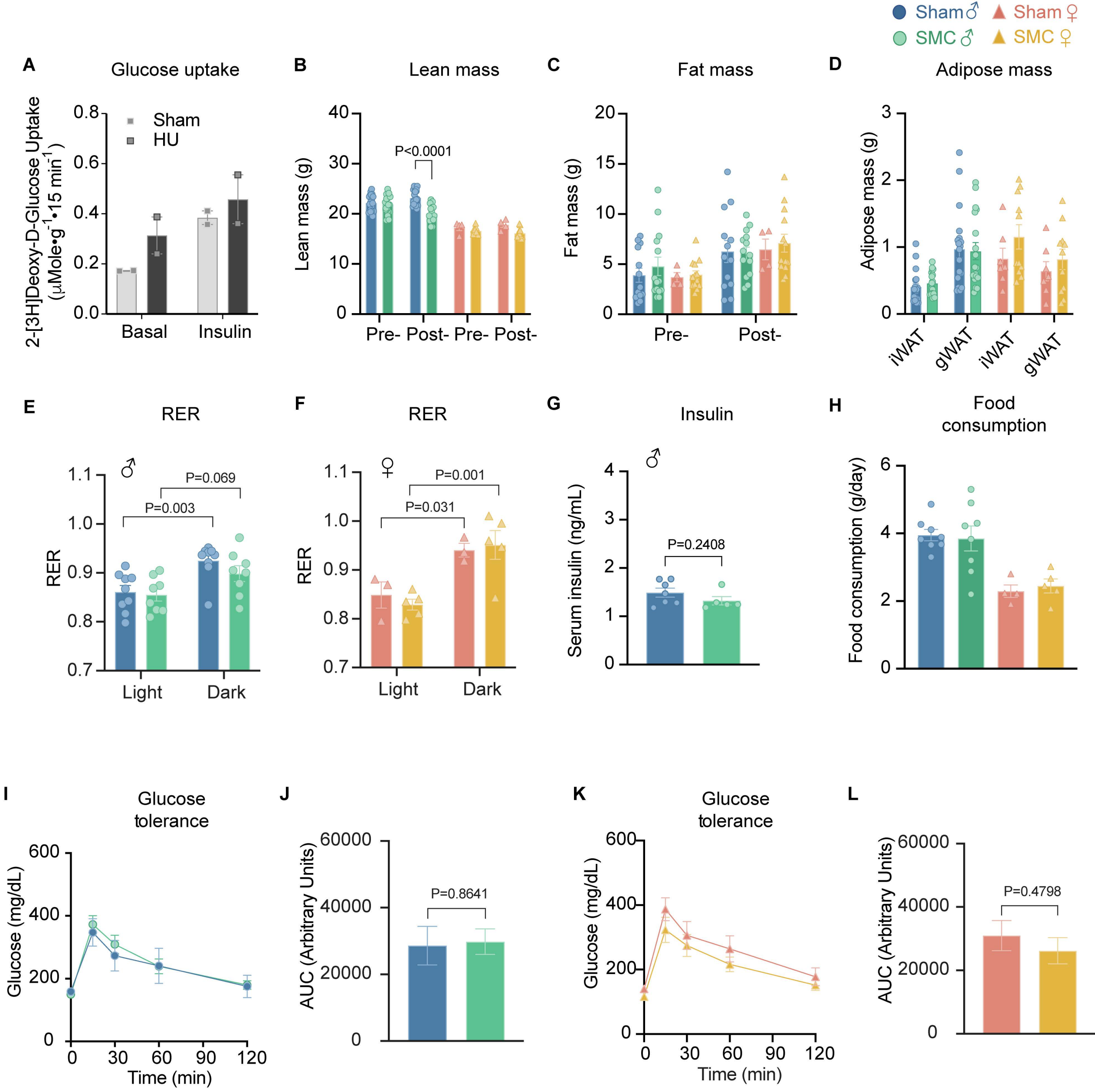

Figure S1. (A) [^{3} H]2-deoxyglucose glucose uptake in soleus muscles of sham and hindlimb unloading (HU) mice (*n* = 2 per group). (B) Lean mass pre- and post-small mouse cage (SMC) intervention by NMR (*n* = 12-15 per group). (C) Fat mass pre- and post-intervention by NMR (*n* = 4-13 per group). (D) Adipose mass of sham and SMC mice (*n* = 7-19 per group). Average respiratory exchange ratio (RER) of sham and SMC male (E) (*n* = 8-9 per group) and female (F) mice (*n* = 3-5 per group). (G) Fasting serum insulin levels in sham and SMC male mice (*n* = 5-7 per group). (H) Food consumption (*n* = 4-8 per group). Glucose tolerance test (GTT) and area under the curve (AUC) of the GTT in sham and SMC male (I-J) (*n* = 6-12 per group) and female (K-L) mice (*n* = 7-13 per group). Data represent mean ± SEM. P-values generated by two-tailed, equal variance, Student's t-test (G, J, L), or by 2-way ANOVA with Tukey's post hoc test (A-F, H, I, K).

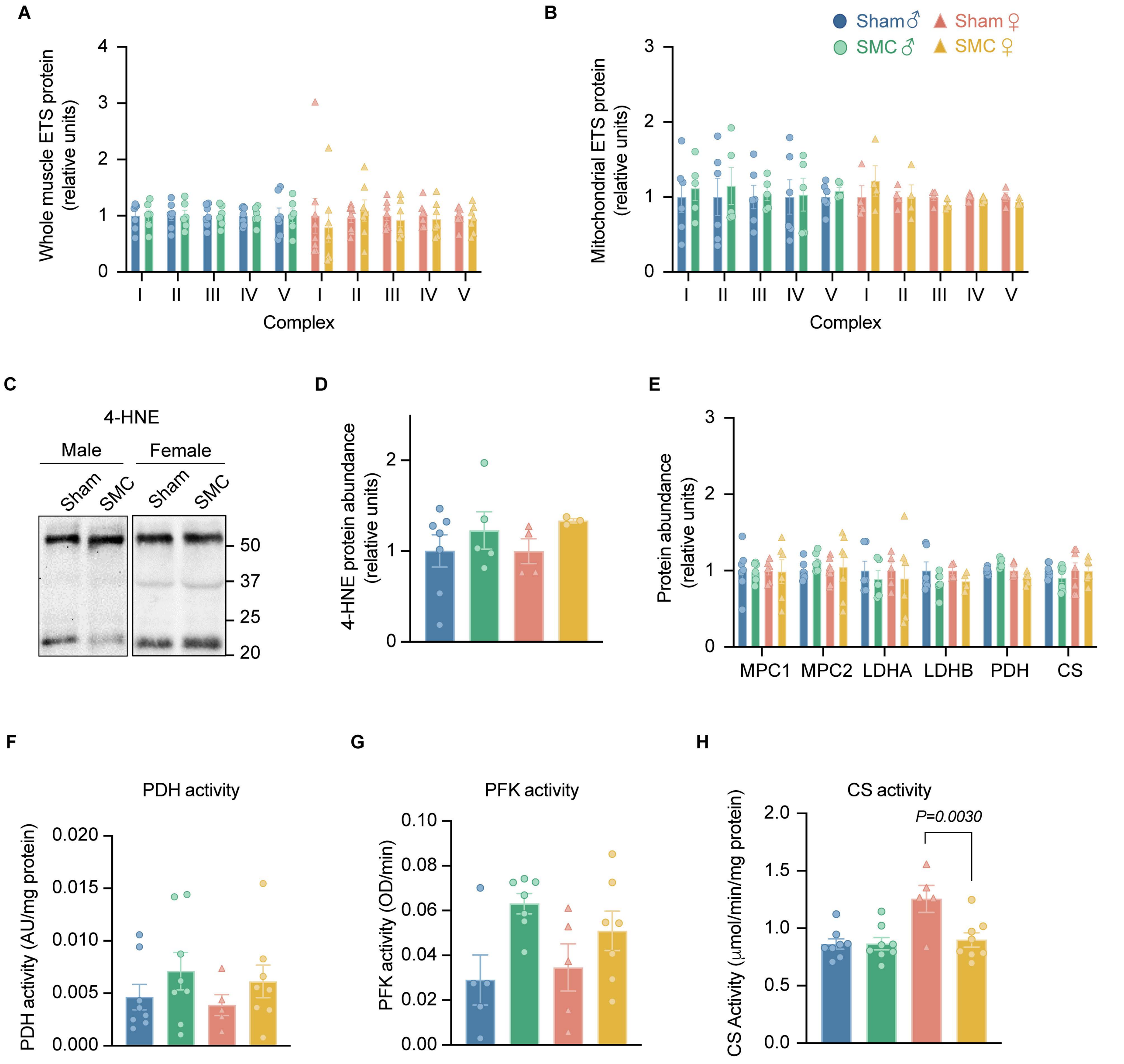

Figure S2. (A) Skeletal muscle respiratory complex quantification (*n* = 5-8 per group). (B) Isolated muscle mitochondria respiratory complex quantification (*n* = 5-6 per group). (C) Representative 4-hydroxynonenal (4-HNE) western blot of whole gastrocnemius muscles of sham and SMC mice (*n* = 5-7 per group). (D) 4-HNE protein quantification (*n* = 5-7 per group). (E) Quantification of western blots of enzymes involved in pyruvate metabolism quantification (*n* = 4-7 per group). (F) Pyruvate dehydrogenase (PDH) activity assay (*n* = 5-8 per group). (G) Phosphofructokinase (PFK) activity assay (*n* = 5-7 per group). (H) Citrate synthase (CS) activity assay (*n* = 5-8 per group). Data represent mean ± SEM. P-values generated by 2-way ANOVA with Tukey's post hoc test (A-B, D-H).

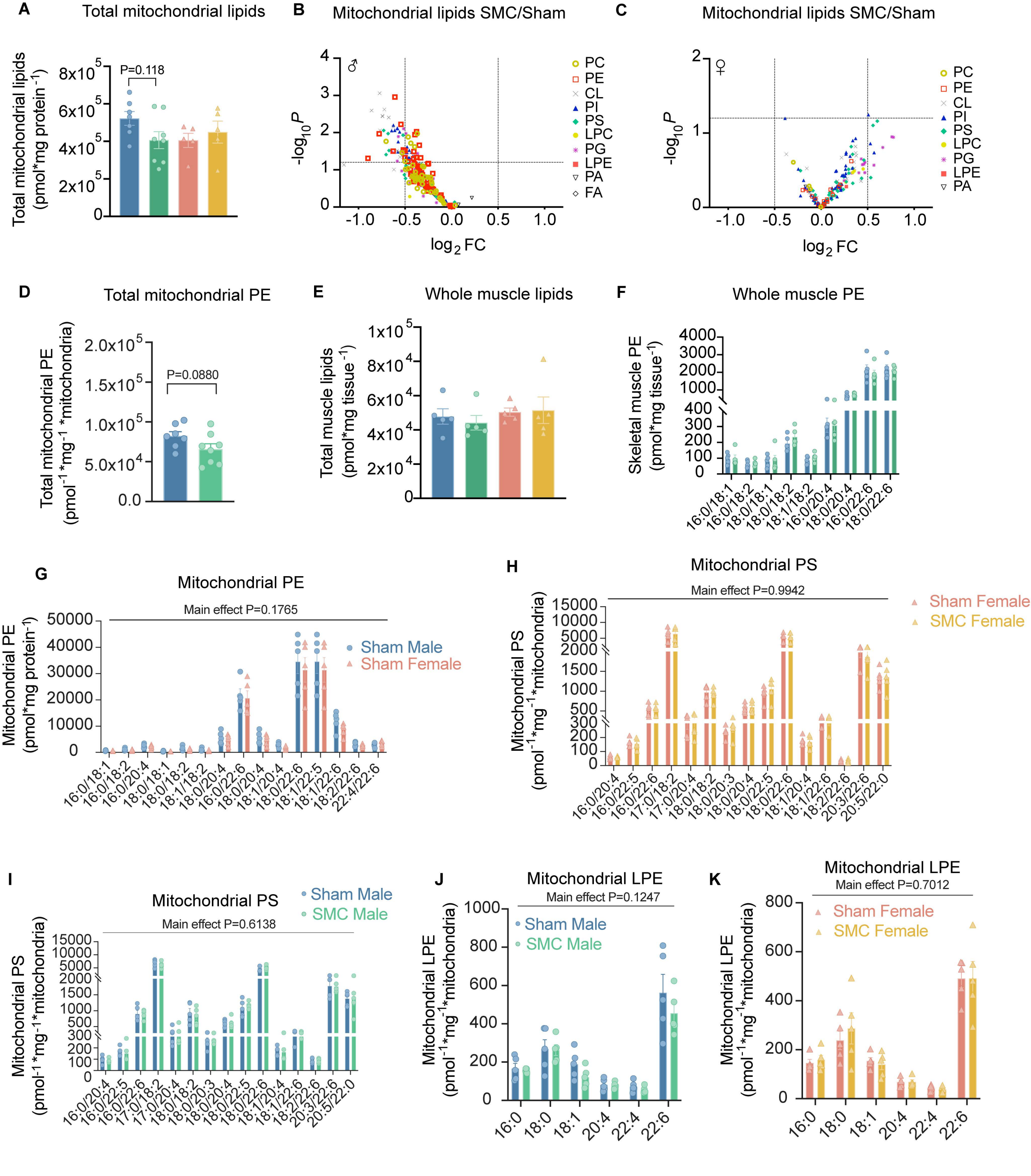

Figure S3. (A) Total skeletal muscle (gastrocnemius) mitochondrial lipids between male sham and SMC mice (n = 7-8 per group) and female sham and SMC mice (n = 5 per group). Volcano plot showing changes in muscle mitochondrial lipids between male (B) and female (C) sham and SMC mice (n = 8 per group). (D) Total mitochondrial phosphatidylethanolamine (PE). Whole skeletal muscle (not isolated mitochondria) total lipids (E) (n = 5 per group) and PE (F) (n = 7-8 per group). (G) Skeletal muscle mitochondrial PE levels in sham male and female mice (n = 5 per group). Skeletal muscle mitochondrial phosphatidylserine (PS) levels in sham and SMC female (H) and male mice (I) (n = 5 per group). Skeletal muscle mitochondrial lysophosphatidylethanolamine (LPE) levels in sham and SMC male (J) and female mice (K) (n = 5 per group). MPC1: mitochondrial pyruvate carrier complex 1; MPC2; mitochondrial pyruvate carrier complex 2; PDH: pyruvate dehydrogenase; LDHA: lactate dehydrogenase isoform A; LDHB: lactate dehydrogenase isoform B; CS: citrate synthetase. Data represent mean ± SEM. P-values generated by Student's t-test (D) and 2-way ANOVA with Tukey's post hoc test (A and E-K).

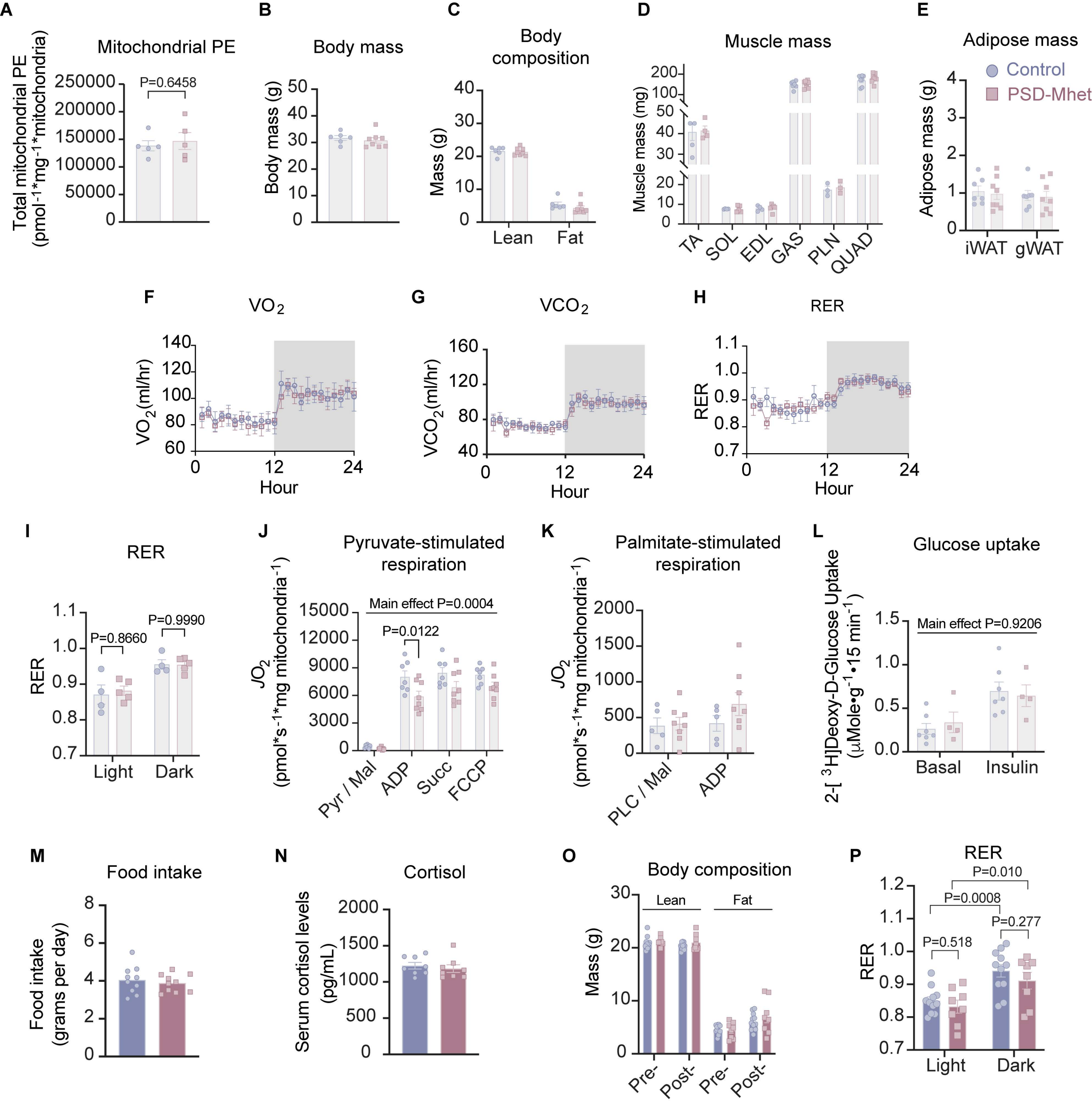

Figure S4. (A) Total skeletal muscle mitochondrial PE levels from gastrocnemius muscles of SMC control and PSD-Mhet mice (*n* = 5 per group). (B) Body mass of sham control and PSD-Mhet (PSD muscle-specific heterozygous knockout) mice (*n* = 6-8 per group). (C) Lean and fat mass by NMR of sham control and PSD-Mhet mice (*n* = 6-8 per group). (D) Skeletal muscle mass of sham control and PSD-Mhet mice (*n* = 3-8 per group). (E) Adipose tissue mass of sham control and PSD-Mhet mice (*n* = 7-8 per group). (F) VO2 of sham control and PSD-Mhet mice (*n* = 4-5 per group). (G) VCO_2 of sham control and PSD-Mhet mice (*n* = 4-5 per group). (H-I) Respiratory exchange ratio (RER) of sham control and PSD-Mhet mice (*n* = 4-5 per group). (J) O_2 utilization in isolated mitochondria measured in the presence of 2 mM ADP (adenosine diphosphate), 0.5 mM malate, 5 mM pyruvate (Pyr), 10 mM succinate (Succ), 1 μM carbonyl cyanide-*p*-trifluoromethoxyphenylhydrazone (FCCP) of sham control and PSD-Mhet mice (*n* = 7-8 per group). (K) O_2 utilization in isolated mitochondria measured in the presence of 2 mM ADP, 0.5 mM malate (Mal), 0.02 mM palmitoyl-L-carnitine (PLC) of sham control and PSD-Mhet mice (*n* = 5-8 per group). (L) [^{3}H]2-deoxyglucose glucose uptake in soleus muscles of sham control and PSD-Mhet mice with or without 200μU/mL of insulin (*n* = 4-7 per group). (M) Average food intake per day throughout SMC intervention (*n* = 10 per group). (N) Serum cortisol levels after SMC intervention (*n* = 8 per group). (O) Lean and fat mass by NMR of SMC Control and SMC PSD-Mhet mice pre- and post-intervention (*n* = 8-12 per group). (P) Average RER between SMC Control and PSD-Mhet mice (*n* = 8-11 per group). TA: tibialis anterior; SOL: soleus; EDL: extensor digitorum longus; PLN: plantaris; GAS: gastrocnemius; QUAD: quadriceps; iWAT: inguinal white adipose tissue; gWAT: gonadal white adipose tissue. Data are all from male control and PSD-Mhet mice. Data represent mean ± SEM. P-values generated by two-tailed, equal variance, Student's t-test (A, B, M, N), or by 2-way ANOVA with Tukey's post hoc test (C-L, O, P).

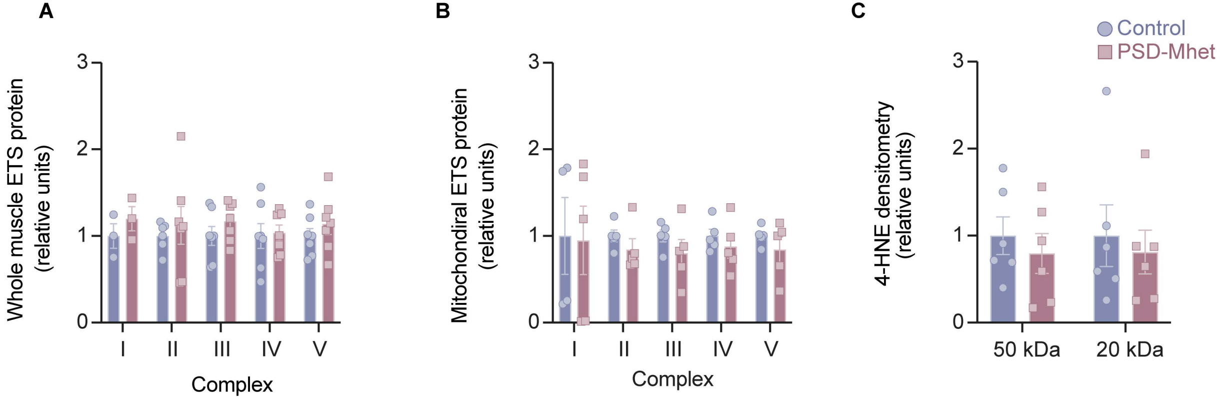

Figure S5. (A) Whole muscle tissue respiratory complex protein quantification (*n* = 4-7 per group). (B) Respiratory complex protein quantification in isolated mitochondria (*n* = 5 per group). (C) 4-hydroxynonenal (4-HNE) protein quantification (*n* = 6 per group). Data are all from male control and PSD-Mhet mice. Data represent mean ± SEM. P-values generated by 2-way ANOVA with Tukey's post hoc test (A-C).

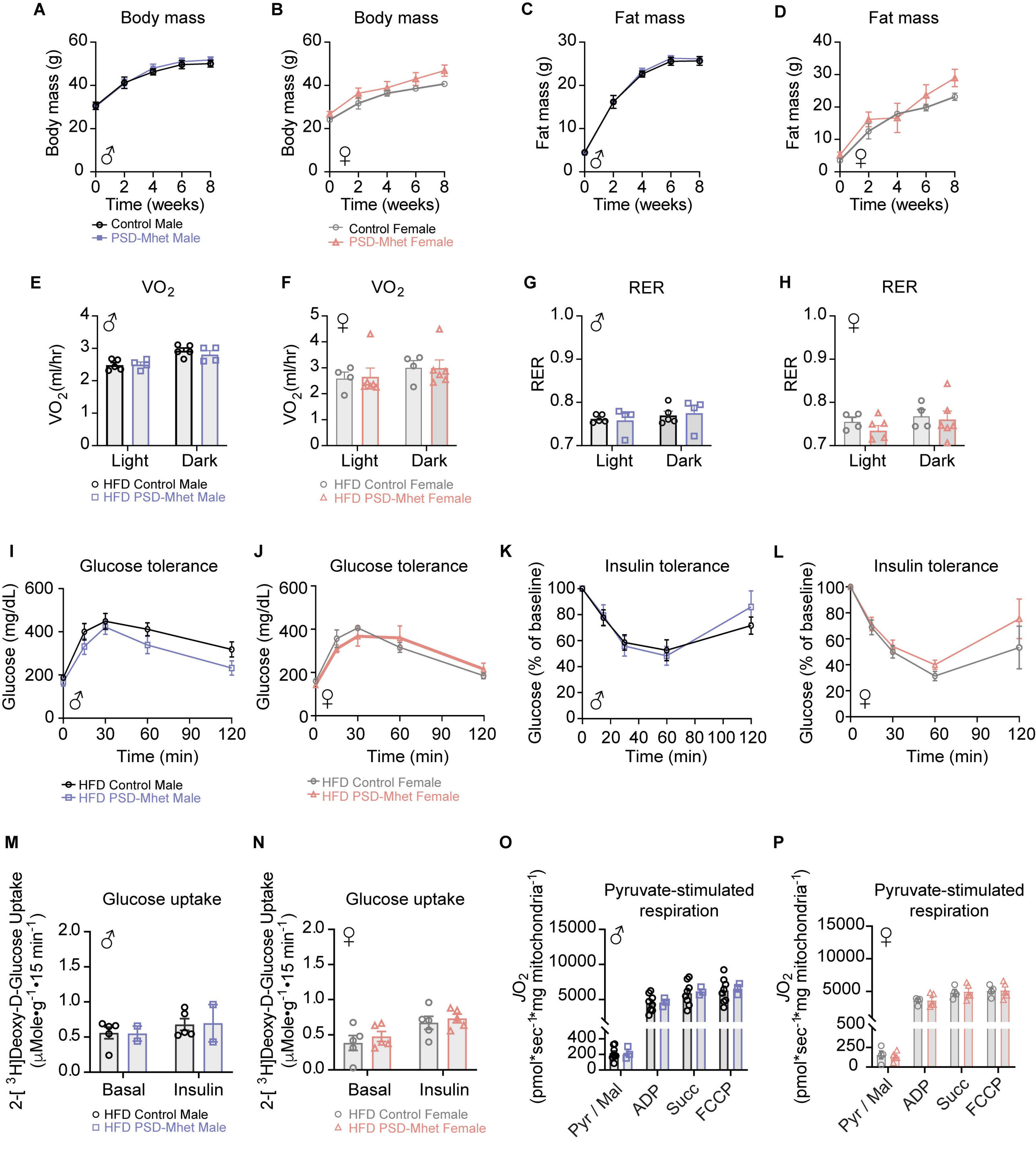

Figure S6. (A-D) Body mass and fat mass of high-fat diet fed control and PSD-Mhet (PSD muscle-specific heterozygous knockout) mice (n = 4-7 per group). (E-F) VO2 of high-fat diet fed control and PSD-Mhet mice (n = 4-5 per group). (F) VCO_2 of sham control and PSD-Mhet mice (n = 4-5 per group). (G-H) Respiratory exchange ratio (RER) of high-fat diet fed control and PSD-Mhet mice (n = 4-5 per group). (I-J) Glucose tolerance test of high-fat diet fed control and PSD-Mhet mice (n = 4-9 per group). (K-L) Insulin tolerance test of high-fat diet fed control and PSD-Mhet mice (n = 6-11 per group). (M-N) [^{3}H]2-deoxyglucose glucose uptake in soleus muscles of high-fat diet fed control and PSD-Mhet mice with or without 200μU/mL of insulin (n = 2-5 per group). (O-P) O_2 utilization in isolated mitochondria measured in the presence of 2 mM ADP (adenosine diphosphate), 0.5 mM malate, 5 mM pyruvate (Pyr), 10 mM succinate (Succ), 1 μM carbonyl cyanide-*p*-trifluoromethoxyphenylhydrazone (FCCP) of sham control and PSD-Mhet mice (n = 3-8 per group). Data represent mean ± SEM. 2-way ANOVA with Tukey's post hoc test for all panels.

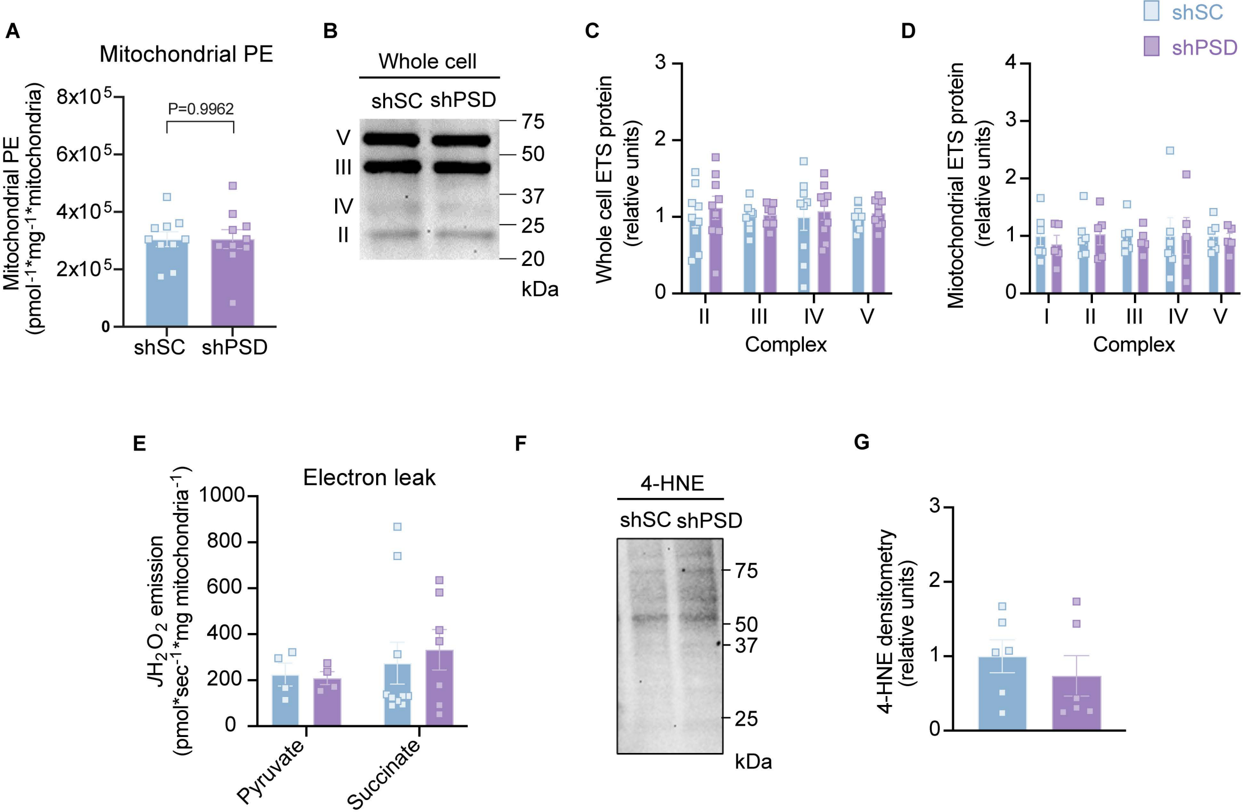

Figure S7. (A) Total mitochondrial PE levels in scrambled (shSC) and PSD knockdown myotubes (shPSD) (*n* = 10 per group). (B) Representative western blot of whole cell respiratory complex proteins between shSC and shPSD cells (*n = 9* per group). (C) Whole cell respiratory complex western blot quantification of (B) (*n = 9* per group). (D) Respiratory complex protein quantification in isolated mitochondria (*n* = 5-6 per group). (E) Hydrogen peroxide (H_2O_2) emission in isolated mitochondria from shSC and shPSD myotubes stimulated with succinate or pyruvate and auranofin (*n* = 4-10 per group). (F) Representative western blot of whole cell 4-hydroxynonenal (4-HNE) (*n* = 6 per group). (G) Quantification of (F) (*n* = 6 per group). Data represent mean ± SEM. P-values generated by two-tailed, equal variance, Student's t-test (A, G) or by 2-way ANOVA with Tukey's post hoc test (C-E).

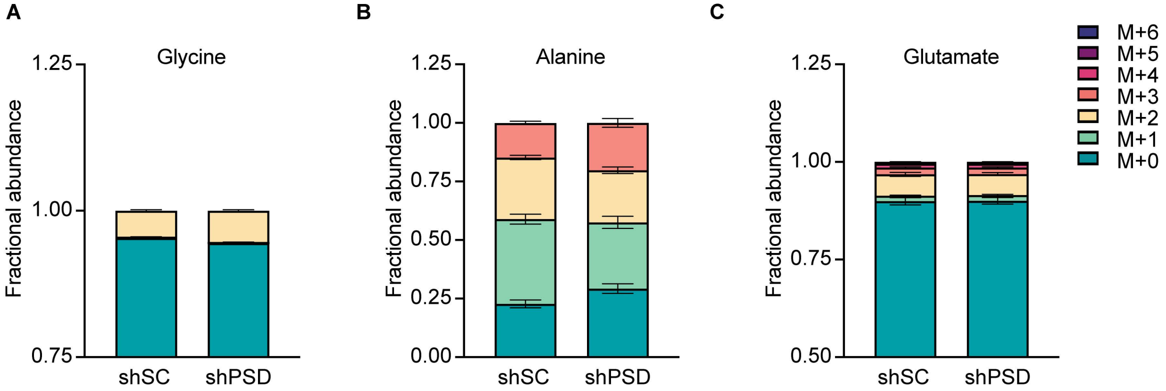

Figure S8. Isotopic labeling pattern in scrambled control (shSC) and PSD knockdown (shPSD) C2C12 myotubes of (A) glycine, (B) alanine, (C) glutamate (*n* = 4-5 per group). Data represent mean ± SEM.

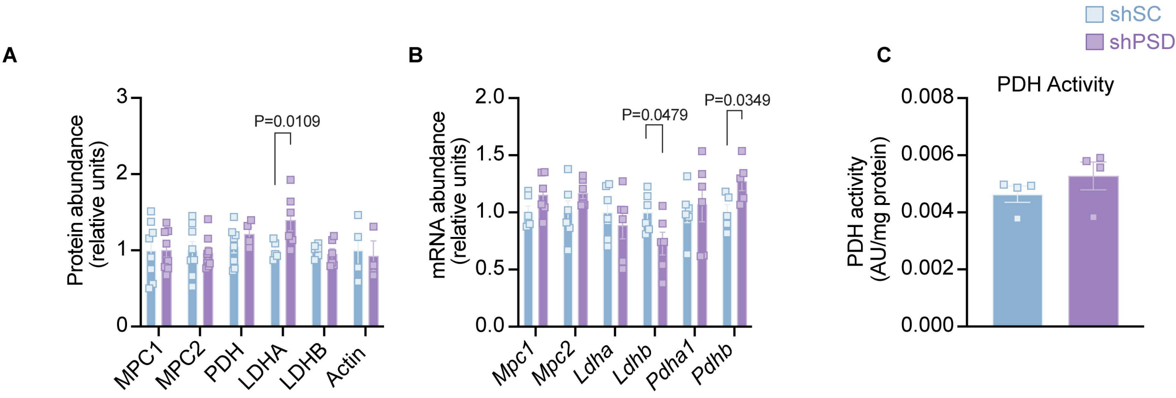

Figure S9. (A) Western blot quantification of MPC1, MPC2, PDH, LDHA, LDHB, and actin protein abundances (n = 4-8 per group). (B) Relative mRNA abundances of genes encoding for proteins in (A) (n = 6 per group). (C) PDH activity (n = 4 per group). MPC1: mitochondrial pyruvate carrier complex 1; MPC2; mitochondrial pyruvate carrier complex 2; PDH: pyruvate dehydrogenase; LDHA: lactate dehydrogenase isoform A; LDHB: lactate dehydrogenase isoform B. *Mpc1*: mitochondrial pyruvate carrier complex 1; *Mpc2*; mitochondrial pyruvate carrier complex 2; *Pdha1, Pdhb*: pyruvate dehydrogenase; *Ldha*: lactate dehydrogenase isoform A; *Ldhb*: lactate dehydrogenase isoform B. Data represent mean ± SEM. by two-tailed, equal variance, Student's t-test (A-C).

Table S1: Primers used for quantitative RT-PCR

Gene	Species	F/R	Sequence (5'→3')
L32	Mouse	F	TTCCTGGTCCACAATGTCAA
		R	GGCTTTTCGGTTCTTAGAGGA
Pisd	Mouse	F	TAC AGG GAA CGG AAG CTT GA
		R	ACG CGT TGG CAC AGA TTT AT
Mpc1	Mouse	F	CTCCAGAGATTATCAGTGGGCG
		R	GAGCTACTTCGTTTGTTACATGGC
Mpc2	Mouse	F	CTCAGTCCACTGTGTTGATGGC
		R	ATCCGAAACAGCTGAGAGGCTC
Ldha	Mouse	F	ACGCAGACAAGGAGCAGTGGAA
		R	ATGCTCTCAGCCAAGTCTGCCA
Ldhb	Mouse	F	CCTCAGATCGTCAAGTACAGCC
		R	ATCCGCTTCCAATCACACGGTG
Pdha1	Mouse	F	GTGAGAACAACCGCTATGGCATG
		R	CGCAAACTTTGTTGCCTCTCGG
Pdhb	Mouse	F	CATCTCGTGACTGTGGAAGGAG
		R	ACATCAGCACCAGTGACACGCA
