# Supplementary material for: Sedentary behavior in mice induces metabolic inflexibility by suppressing skeletal muscle pyruvate metabolism
Source: J Clin Invest. 2024 Apr 23;134(11):e167371. doi: 10.1172/JCI167371 (PMC11142742; doi:10.1172/JCI167371)

Uncropped Western Blot images. Individual panels show uncropped western blots for corresponding figures. Boxed region represents representative area reported

Full unedited blot for Figure 1L

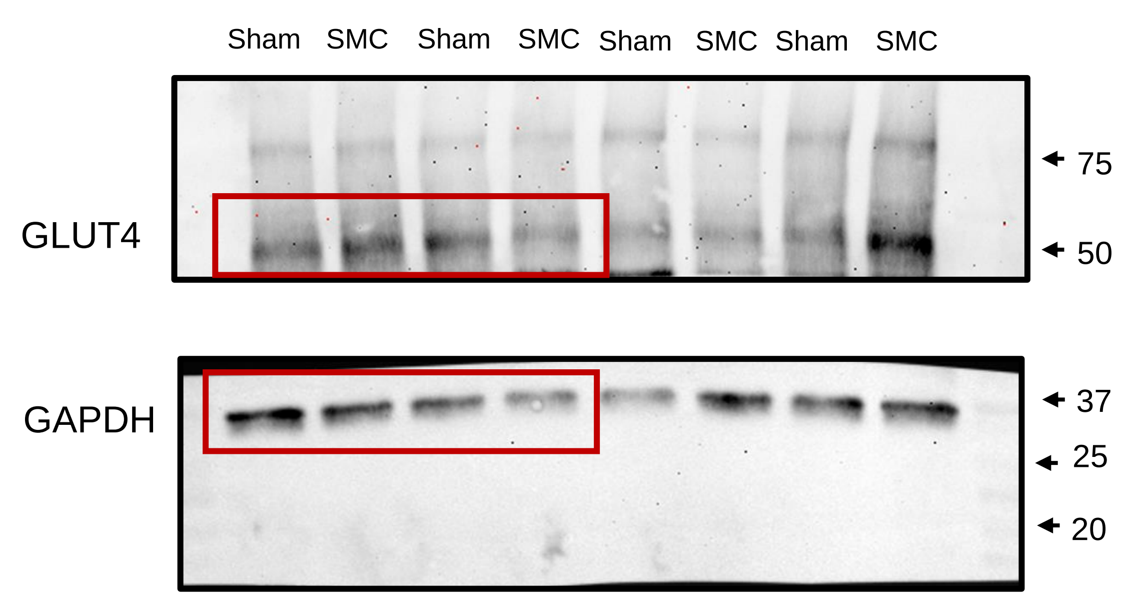

Full unedited blot for Figure 2B – OXPHOS – whole tissue

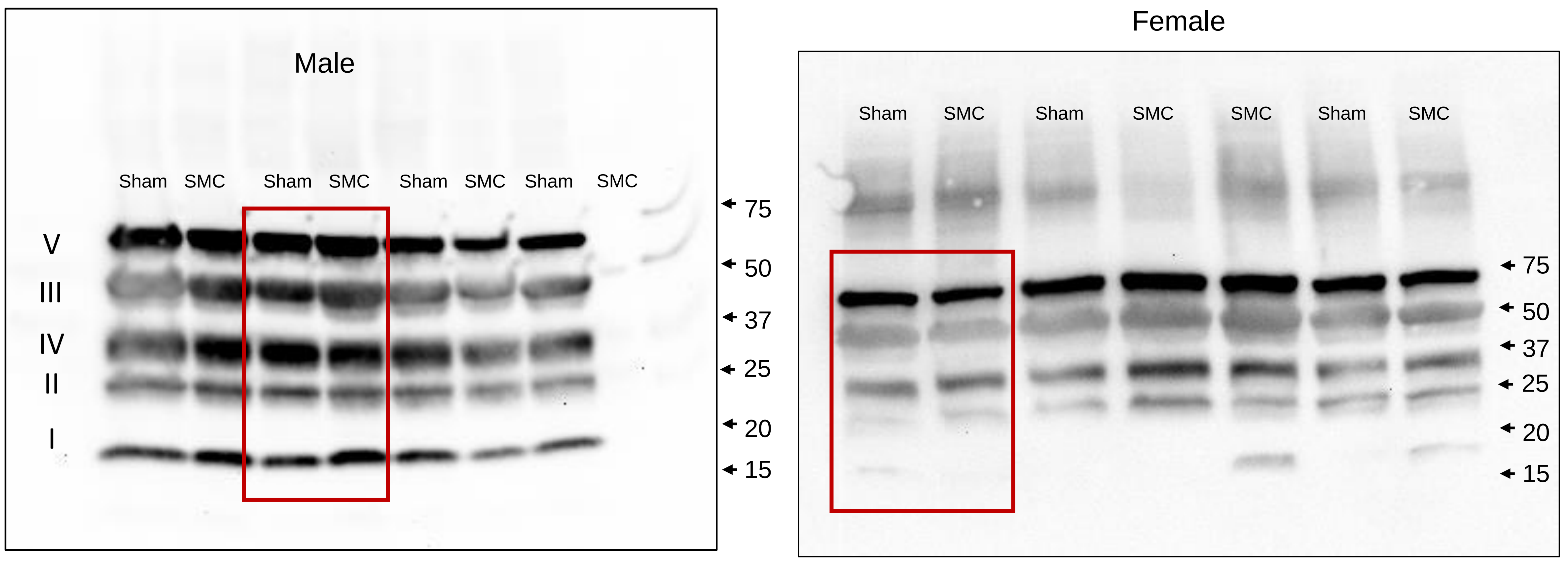

Full unedited blot for Figure 2F – OXPHOS isolated mitochondria

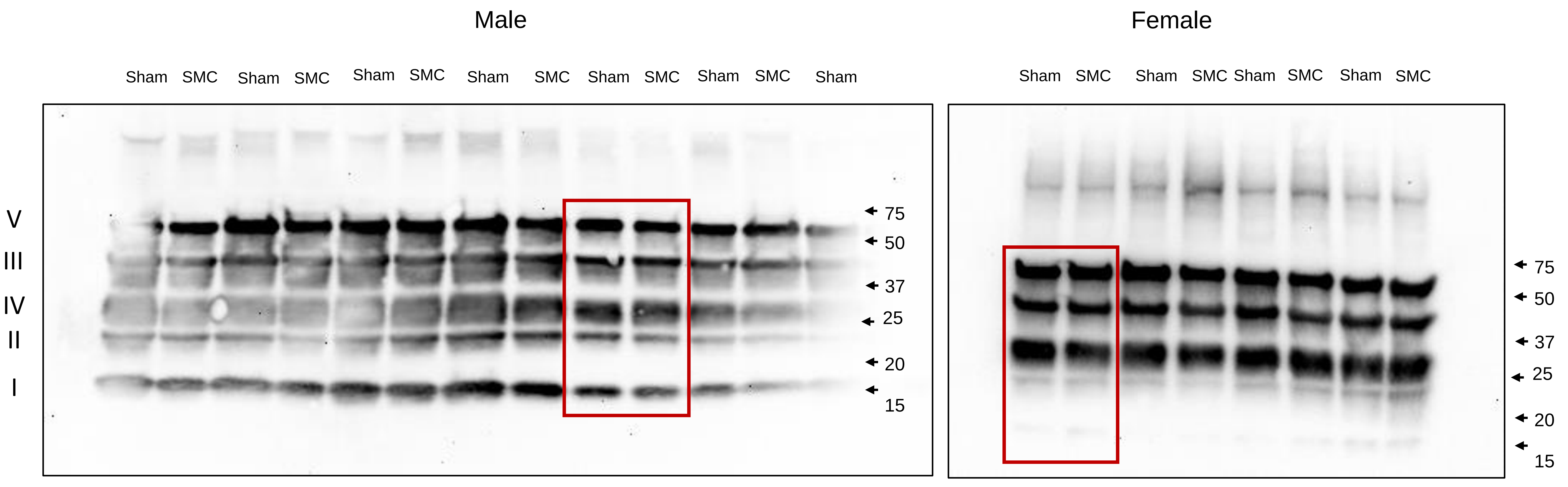

Full unedited blot for Figure S2C – 4HNE

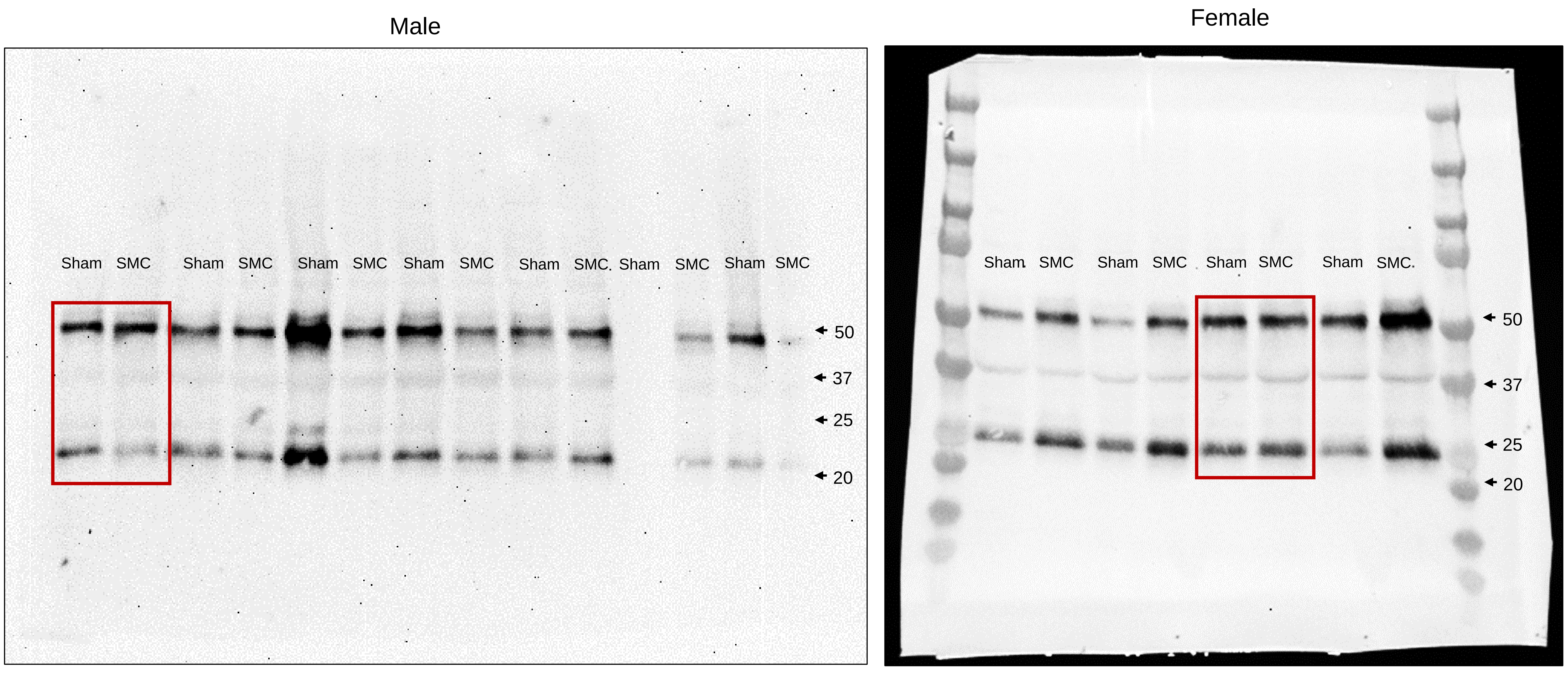

Uncropped Western Blot images. Individual panels show uncropped western blots for corresponding figures. Boxed region represents representative area reported

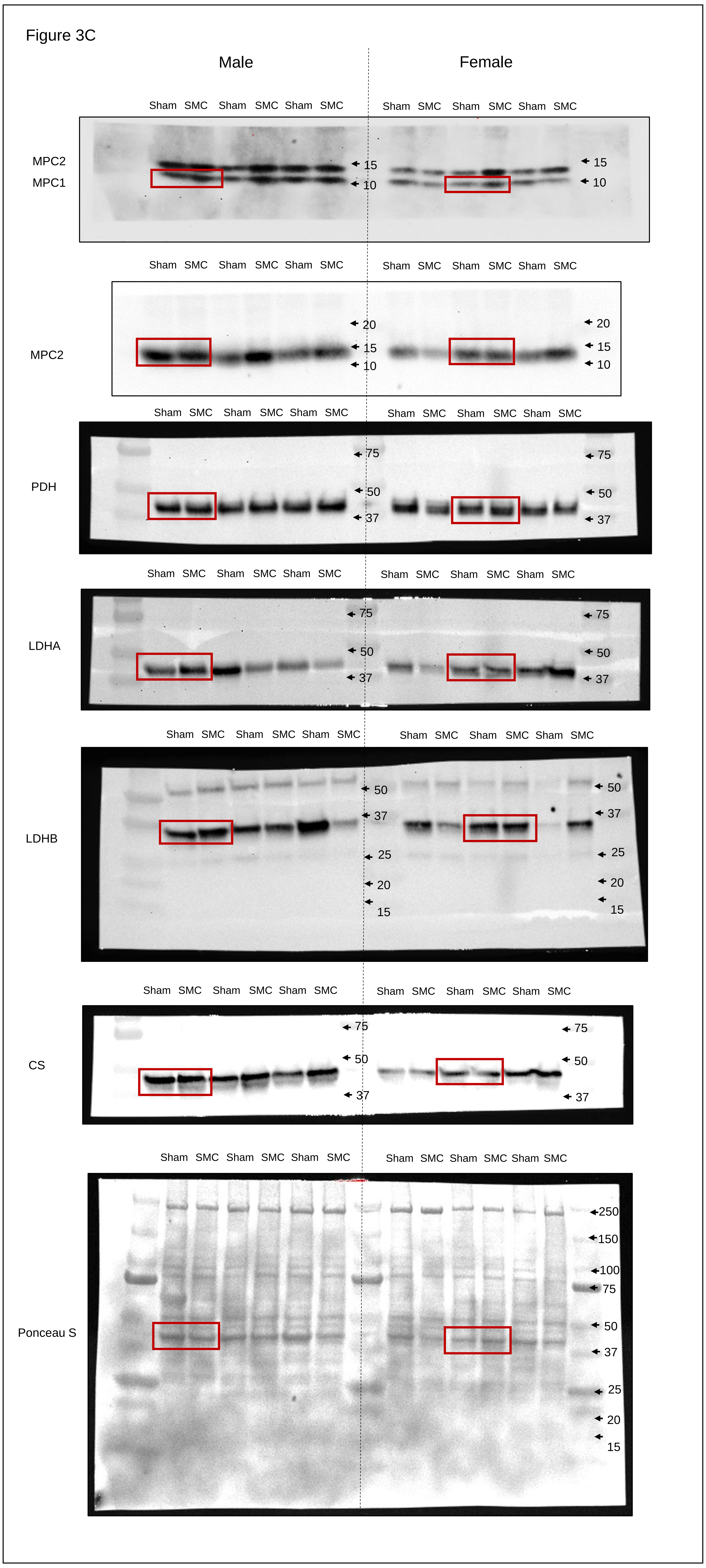

Uncropped Western Blot images. Individual panels show uncropped western blots for corresponding figures. Boxed region represents representative area reported

Full unedited blot for Figure 5A - OXPHOS

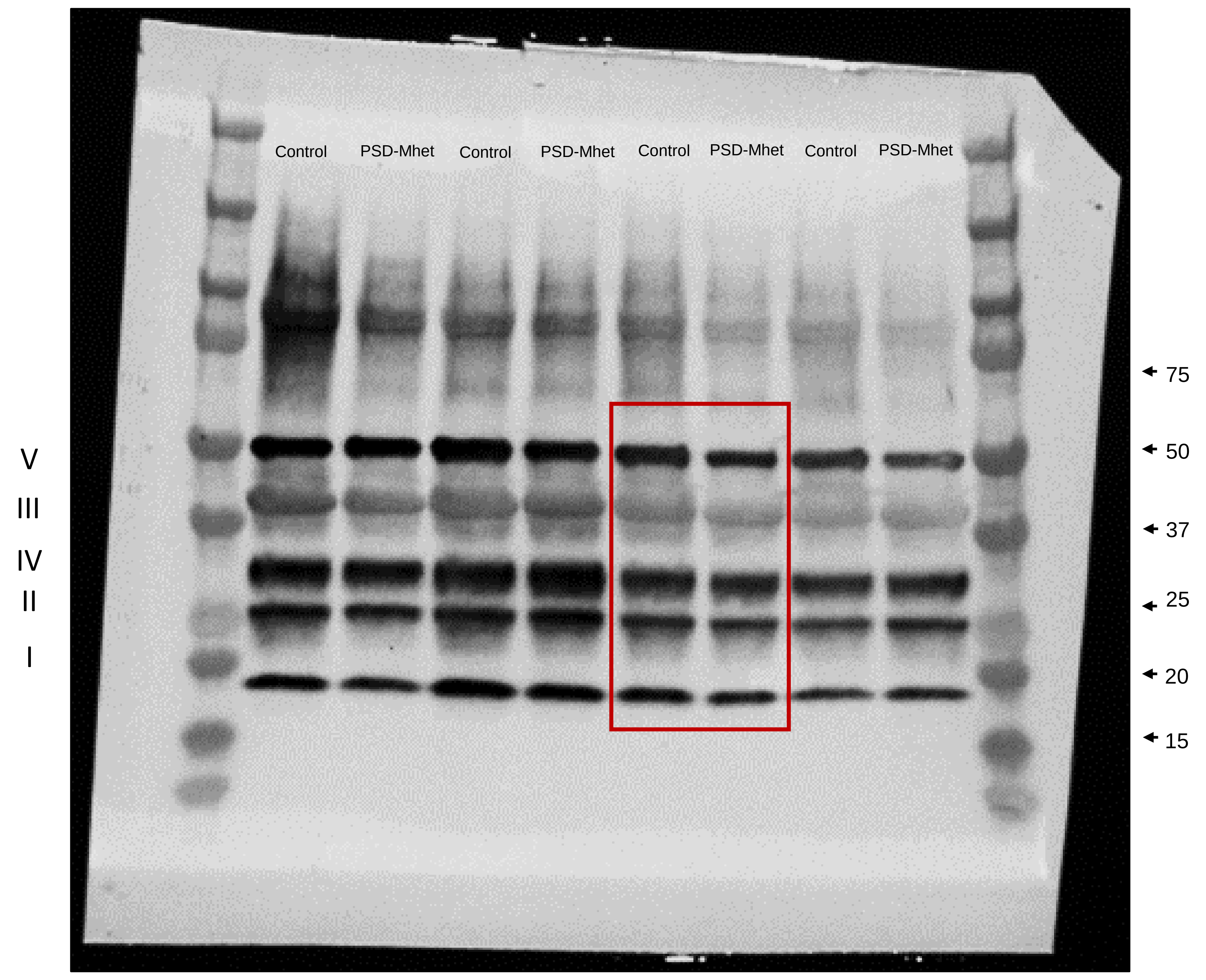

Full unedited blot for Figure 5E - OXPHOS

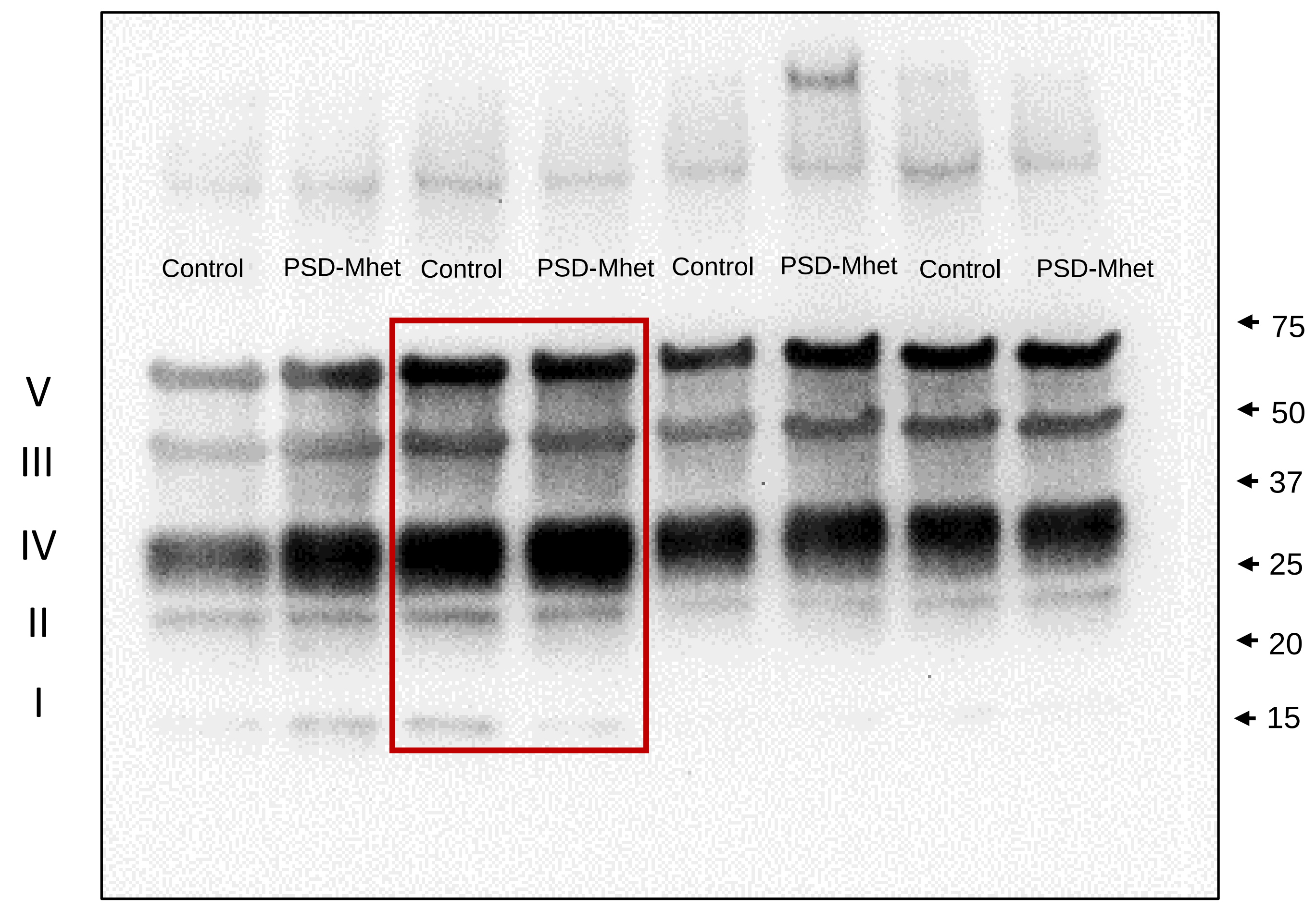

Full unedited blot for Figure 5G – 4HNE

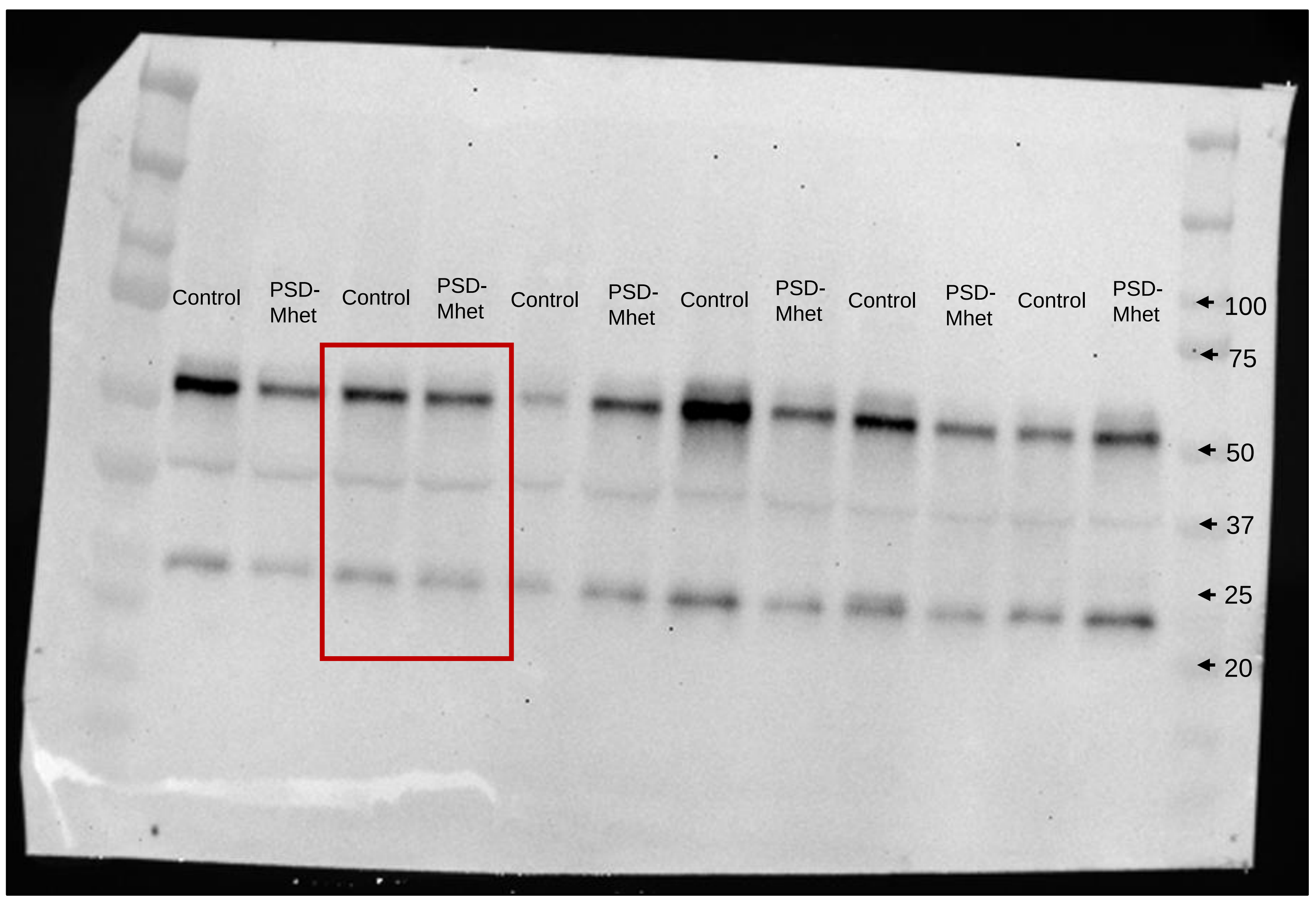

Uncropped Western Blot images. Individual panels show uncropped western blots for corresponding figures. Boxed region represents representative area reported

Full unedited blot for Figure 6F – OXPHOS

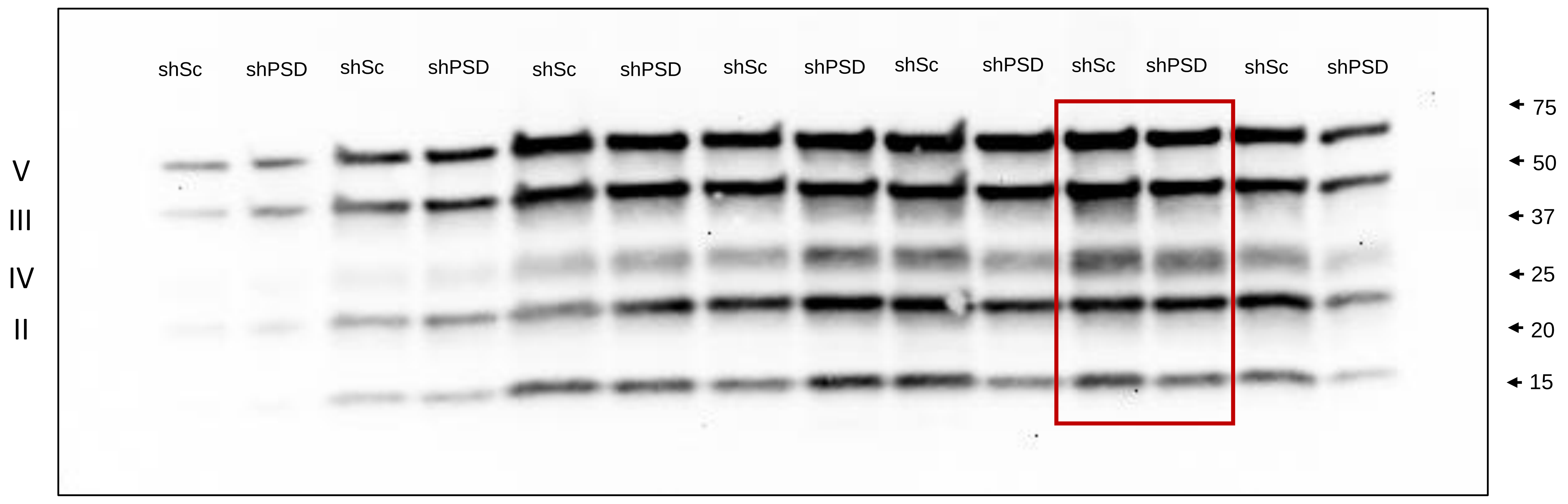

Full unedited blot for Figure S7B – OXPHOS

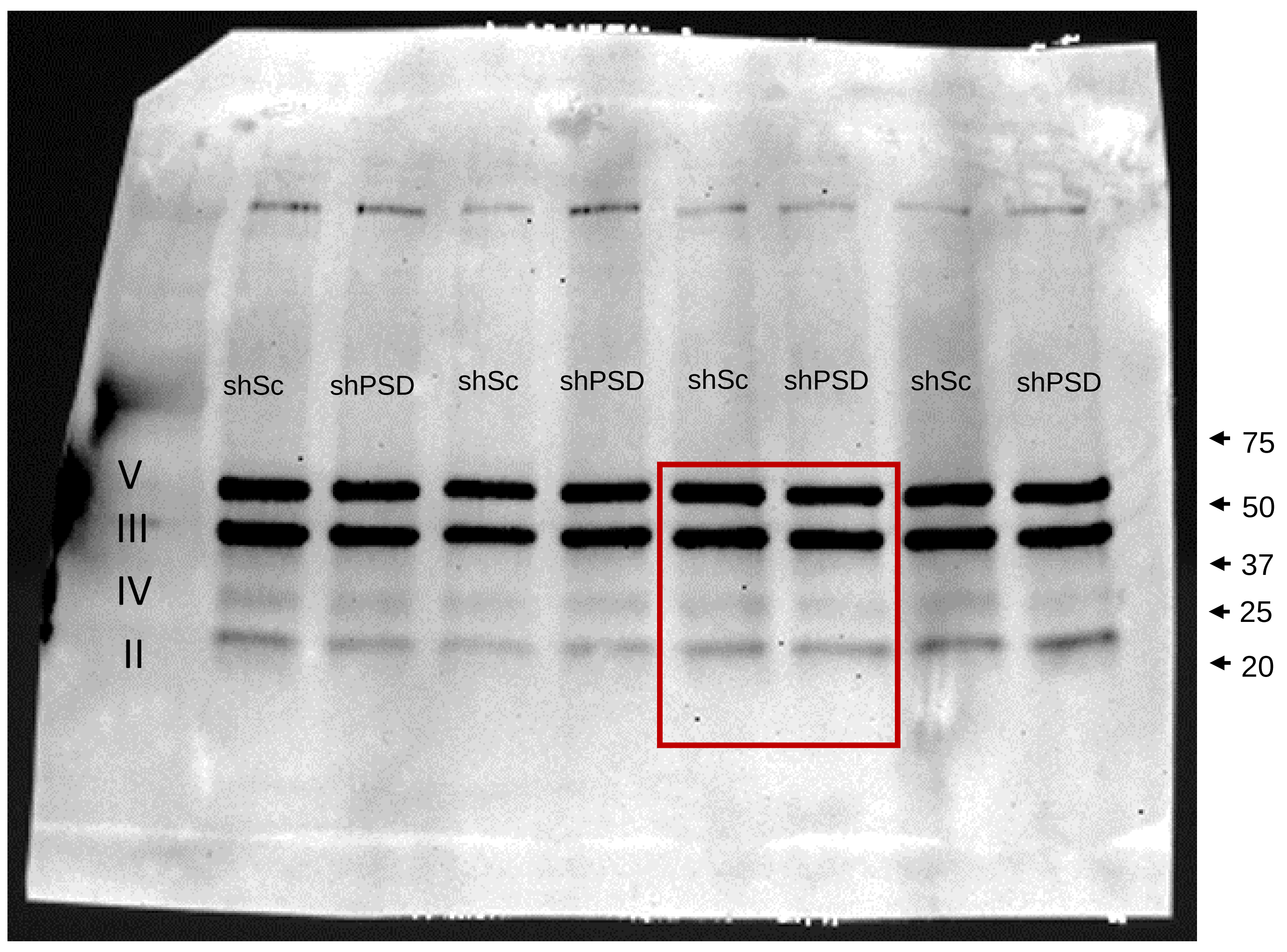

Full unedited blot for Figure S7F – 4HNE

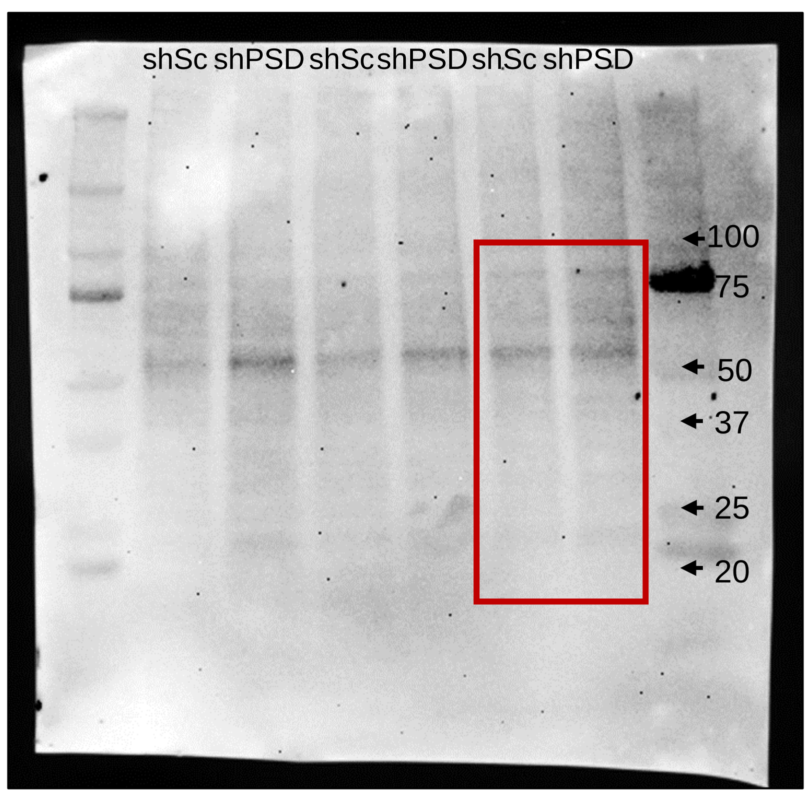

Full unedited blot for Figure 8B

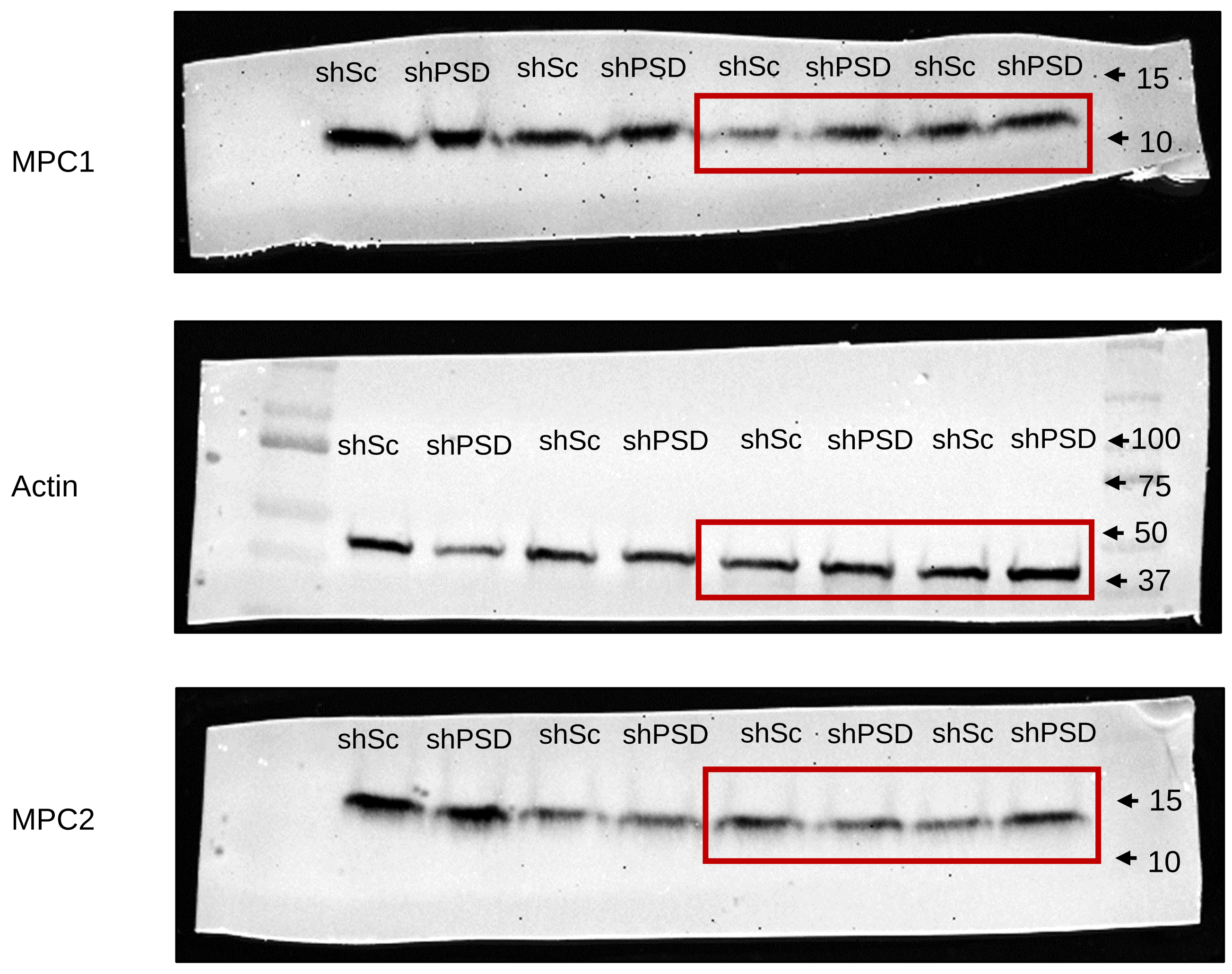

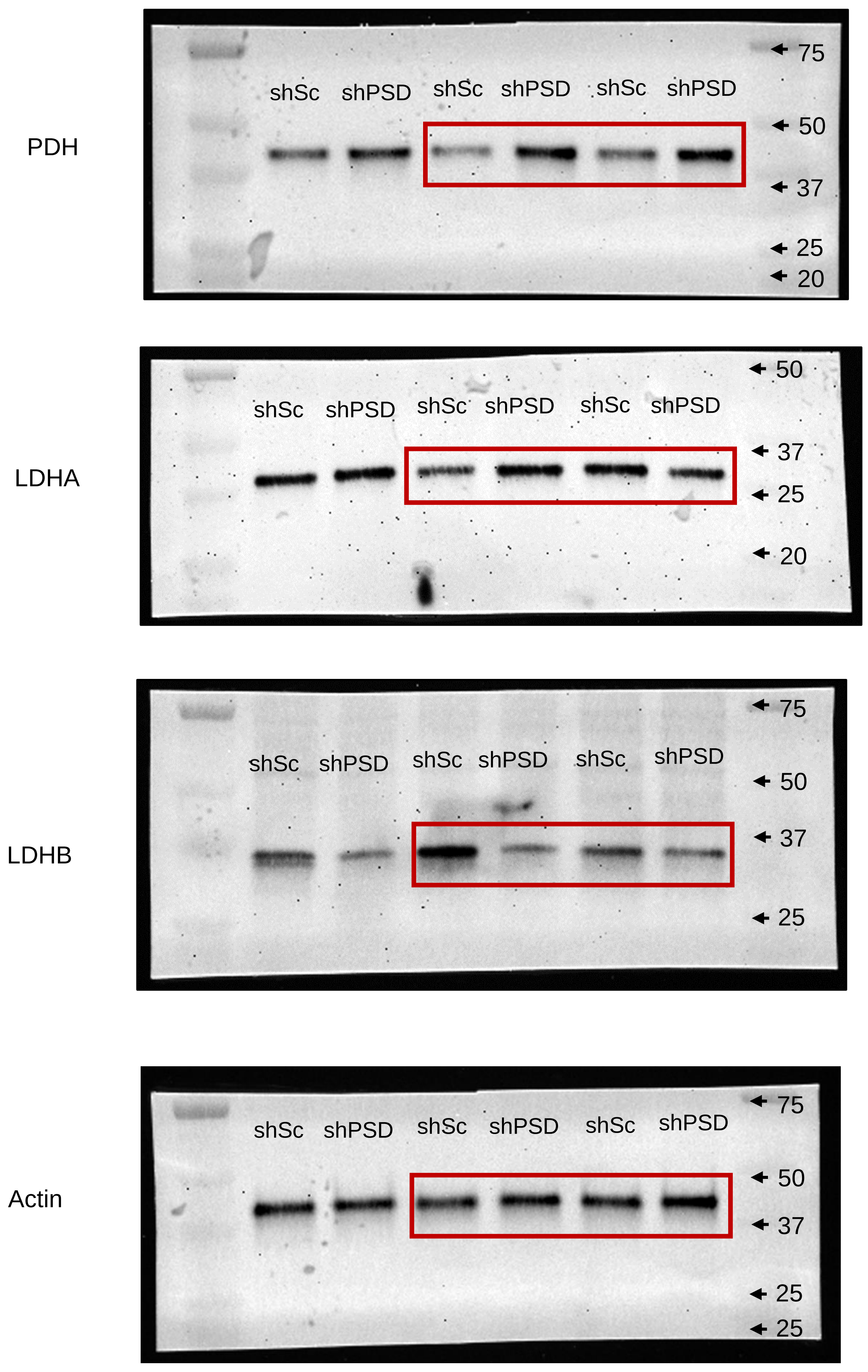

Supplement: Unedited blot and gel images [file jci-134-167371-s245.pdf]
